# Covariates in population pharmacokinetic studies of critically ill adults receiving β-lactam antimicrobials: a systematic review and narrative synthesis

**DOI:** 10.1093/jacamr/dlae030

**Published:** 2024-02-26

**Authors:** Jan Hansel, Fahmida Mannan, Rebecca Robey, Mary Kumarendran, Siân Bladon, Alexander G Mathioudakis, Kayode Ogungbenro, Paul Dark, Timothy W Felton

**Affiliations:** Division of Immunology, Immunity to Infection and Respiratory Medicine, School of Biological Sciences, University of Manchester, Oxford Road, Manchester M13 9PL, UK; Acute Intensive Care Unit, Wythenshawe Hospital, Manchester University NHS Foundation Trust, Southmoor Road, Wythenshawe, Manchester M23 9LT, UK; Division of Cardiovascular Sciences, School of Medical Sciences, University of Manchester, Oxford Road, Manchester M13 9PL, UK; Division of Immunology, Immunity to Infection and Respiratory Medicine, School of Biological Sciences, University of Manchester, Oxford Road, Manchester M13 9PL, UK; Acute Intensive Care Unit, Wythenshawe Hospital, Manchester University NHS Foundation Trust, Southmoor Road, Wythenshawe, Manchester M23 9LT, UK; Division of Informatics, Imaging & Data Sciences, School of Health Sciences, University of Manchester, Oxford Road, Manchester M13 9PL, UK; Division of Immunology, Immunity to Infection and Respiratory Medicine, School of Biological Sciences, University of Manchester, Oxford Road, Manchester M13 9PL, UK; North West Lung Centre, Wythenshawe Hospital, Manchester University NHS Foundation Trust, Southmoor Road, Wythenshawe, Manchester M23 9LT, UK; Division of Pharmacy & Optometry, School of Health Sciences, University of Manchester, Oxford Road, Manchester M13 9PL, UK; Division of Immunology, Immunity to Infection and Respiratory Medicine, School of Biological Sciences, University of Manchester, Oxford Road, Manchester M13 9PL, UK; Critical Care Unit, Northern Care Alliance NHS Foundation Trust, Salford Care Organisation, Greater Manchester M6 8HD, UK; Division of Immunology, Immunity to Infection and Respiratory Medicine, School of Biological Sciences, University of Manchester, Oxford Road, Manchester M13 9PL, UK; Acute Intensive Care Unit, Wythenshawe Hospital, Manchester University NHS Foundation Trust, Southmoor Road, Wythenshawe, Manchester M23 9LT, UK

## Abstract

**Introduction:**

Population pharmacokinetic studies of β-lactam antimicrobials in critically ill patients derive models that inform their dosing. In non-linear mixed-effects modelling, covariates are often used to improve model fit and explain variability. We aimed to investigate which covariates are most commonly assessed and which are found to be significant, along with global patterns of publication.

**Methods:**

We conducted a systematic review, searching MEDLINE, Embase, CENTRAL and Web of Science on 01 March 2023, including studies of critically ill adults receiving β-lactam antimicrobials who underwent blood sampling for population pharmacokinetic studies. We extracted and categorized all reported covariates and assessed reporting quality using the ClinPK checklist.

**Results:**

Our search identified 151 studies with 6018 participants. Most studies reported observational cohorts (120 studies, 80%), with the majority conducted in high-income settings (136 studies, 90%). Of the 1083 identified covariate instances, 237 were unique; the most common categories were patient characteristics (*n* = 404), biomarkers (*n* = 206) and physiological parameters (*n* = 163). Only seven distinct commonly reported covariates (CL_CR_, weight, glomerular filtration rate, diuresis, need for renal replacement, serum albumin and C-reactive protein) were significant more than 20% of the time.

**Conclusions:**

Covariates are most commonly chosen based on biological plausibility, with patient characteristics and biomarkers the most frequently investigated. We developed an openly accessible database of reported covariates to aid investigators with covariate selection when designing population pharmacokinetic studies. Novel covariates, such as sepsis subphenotypes, have not been explored yet, leaving a research gap for future work.

## Introduction

Sepsis is a life-threatening organ dysfunction caused by a dysregulated host response to infection.^1^ With 48.9 million incident cases and 11.0 million deaths worldwide estimated by the Global Burden of Diseases, Injuries, and Risk Factors Study in 2017 alone, its true global impact has historically been difficult to capture and define accurately.^2^ With a pronounced systemic inflammatory response and phenomena such as augmented renal clearance (ARC) commonly associated with critical illness in general and sepsis in particular, patients are often underdosed with antimicrobial agents, even at appropriate formulary-based dosing. An inappropriately low drug concentration is estimated to be attained in 40%–45% of critically ill individuals receiving β-lactam antimicrobials, the most commonly used class of antimicrobial in critical care.^3–6^ There are presently no accessible tools available to help clinicians reliably predict which patients may be at a higher risk of underdosing.

Given the complex interplay between the host (patient), pathogen and antimicrobial drug, underdosing of β-lactams may lead to worse outcomes for at-risk patients with sepsis and septic shock. Furthermore, underdosing has been closely associated with the development of antimicrobial resistance (AMR), which is more likely to develop when the most resistant subpopulations in bacterial colonies are allowed to continue replicating.^7^ To address the hazards of unpredictable dosing, routine therapeutic drug monitoring (TDM) has been advocated for critically ill patients receiving β-lactam antimicrobials.^3^ However, the major drawbacks to routine TDM are 2-fold: specialized equipment and skilled staff are expensive and rarely available outside of regular working hours or in resource-poor settings; and sampling is often performed relatively late in the course of treatment,^8^ likely leading to a lack of evidence of improved outcomes in recent trials.^9,10^

Nevertheless, precision medicine-based approaches in critical care remain an active area for research and care innovation. Model-informed precision dosing (MIPD) integrates TDM and population pharmacokinetics (PK) to predict the PTA using Bayesian methods, and subsequently tailor the dosing to a given patient.^11,12^ Multiple recent trials have evaluated population PK of commonly used agents like meropenem and piperacillin.^13–16^ An important consideration when selecting the population PK models is the need to be representative of the populations they are actually used in. A model derived in Germany may not reliably translate to patients in the UK or the Democratic Republic of the Congo, possibly owing to genetic and non-genetic factors.^17^ Efforts to perform side-by-side comparisons of models to derive the ‘*one model to rule them all’* are consequently likely to result in suboptimal performance when applied to a given population they have not been developed in.

In the context of population PK studies of β-lactam antimicrobials, serial blood samples are taken from a group of patients and drug concentrations measured at various timepoints in the dosing interval. A non-linear mixed-effects (NLME) base model is then fitted to the measured concentrations; both fixed effects (parameters that have the same value between subjects) and random effects (quantification of unexplained variability between subjects and individual observations) are incorporated into the model, with measures of residual variability and variability between individuals accounted for. The model is then incrementally improved by exploring the influence of a range of continuous or categorical clinical covariates: those that improve model fit (significant) are retained, whereas those that do not (non-significant) are discarded. Alternative methods exist for modelling PK, such as physiologically based PK modelling (PBPK) and techniques like allometric scaling to account for weight in untested populations (e.g. paediatrics). Decisions regarding which covariates to test in population PK studies are built on a number of assumptions, with the overwhelming majority being mathematical in nature (e.g. the principle of parsimony and computational efficiency).^18^ However, clinical and biological plausibility need to be considered and should, along with adoption of previously reported covariates for a given agent, inform the prespecified selection of tested covariates when planning prospective studies. This generates a risk of circuitous repeated selection of the same variables, disregarding possible novel covariates emerging from other planes of scientific observation and investigation.

The choice of covariates in critically ill patients has not been previously systematically described. To address this knowledge gap, we conducted a systematic review and narrative synthesis of published and ongoing population PK studies of β-lactam antimicrobials in critically ill adults with the aim of exploring which covariates were most frequently reported, and of these, which were found to significantly improve model fit and were retained in existing published models. We hypothesized that a handful of similar covariates are tested in a sizeable proportion of studies, few of which are ultimately found to be significant and retained in NLME models.

## Methods

This systematic review and narrative synthesis adhered to the Preferred Reporting Items for Systematic reviews and Meta-Analyses 2020 (PRISMA) and the Synthesis without meta-analysis (SWiM) in systematic reviews reporting guidelines.^19,20^ The review was prospectively registered (CRD42017081279).^21^ Systematic review registration, PROSPERO ID: CRD42023397938, registered 02 March 2023 (https://www.crd.york.ac.uk/prospero/display_record.php? RecordID=397938).

The PRISMA and SWiM checklists are presented in Figures S1 and S2 (available as Supplementary data at *JAC-AMR* Online).

### Eligibility criteria

We included studies of critically ill participants admitted to the ICU aged 18 years and older who received β-lactam antimicrobials and had blood samples (serum or plasma) taken for population PK analyses. We included both prospective and retrospective observational studies, randomized controlled trials (RCTs) and retrospective studies that used previously collected datasets if a new PK model was developed as part of the study using these data. We did not impose restrictions on publication. We excluded case reports, case series of fewer than five patients, expert opinions, letters, correspondence and review articles.

### Sources of information

We searched the following databases for relevant studies on 01 March 2023: Cochrane Central Register of Controlled Trials (CENTRAL), MEDLINE via Ovid (inception to 28 February 2023), Embase via Ovid (inception to 28 February 2023) and Web of Science (inception to 28 February 2023). We searched the ClinicalTrials.gov trial register for ongoing studies on 25 June 2023. The search strategies are reported on searchRxiv.org^22–26^ and in Appendix S1. We applied no restrictions on publication status. We included publications that reported study data, and excluded abstracts and conference proceedings.

### Search strategy, study selection and data extraction

Our comprehensive search strategy, including both free search terms and controlled vocabulary, was developed and run by the authors and peer-reviewed by an information specialist. Following deduplication, two members of the author group (J.H., M.K.) performed title and abstract screening, after which we retrieved the full texts of all eligible studies and assessed them for inclusion. We extracted relevant information about baseline study characteristics, antimicrobial, PK and modelling data into a piloted Google Sheets spreadsheet. The data extraction form is presented in Appendix S2. All data were extracted by one of three authors (J.H., F.M., R.R.) and cross-checked by another second author (M.K., J.H., F.M., R.R.). Any disagreements were resolved by a third senior author. The primary outcome of interest for each study was (i) a list of covariates used in NLME models, (ii) their reported significance or non-significance (with significance thresholds assumed at levels reported in each study) and (iii) for those reported to be significant, which parameter they were significant for.

### Risk-of-bias assessment

We assessed the risk of bias in any included RCTs using Risk of Bias 2 (RoB2). As most population PK studies are not interventional in nature and employ an observational single-cohort design, we assessed the reporting quality of all included studies using the 24-item ClinPK checklist from the Reporting Guidelines for Clinical Pharmacokinetic Studies proposed by Kanji *et al.*^27^ Each study report was assessed by two authors independently, with any disagreements resolved by a third senior author. RoB2 graphs were generated using the *robvis* package in R.^28^ We were not able to formally assess reporting bias using funnel plots or statistical testing for small-study effects as no meta-analysis was undertaken.

### Synthesis methods

Statistical analysis was performed using the software package R (version 4.2.3). A statistician independent of the study team reviewed the data extraction table and the statistical analysis plan. No prespecified power calculations were performed as we did not plan to undertake significance testing or inferential statistics with quantitative data. Summary statistics of frequencies are reported in tables with percentages, categorized according to study design. Tabulated frequencies are presented as box-plots, heatmaps and sunburst plots when appropriate.

We categorized covariates according to a preplanned bespoke classification, based on commonly identified patterns in a preliminary literature review conducted by the authors. The following categories were used: patient characteristics; physiological parameters; biomarkers; vital signs; organ dysfunction/support; disease severity score; extracorporeal parameters; and ‘other’. Following extraction of raw covariate descriptions, covariates were deduplicated and thematically grouped. In light of the scoping nature of this review, no formal sensitivity or subgroup analyses were prespecified for the assessment of heterogeneity.

## Results

### Characteristics of included studies

**Figure 1.**
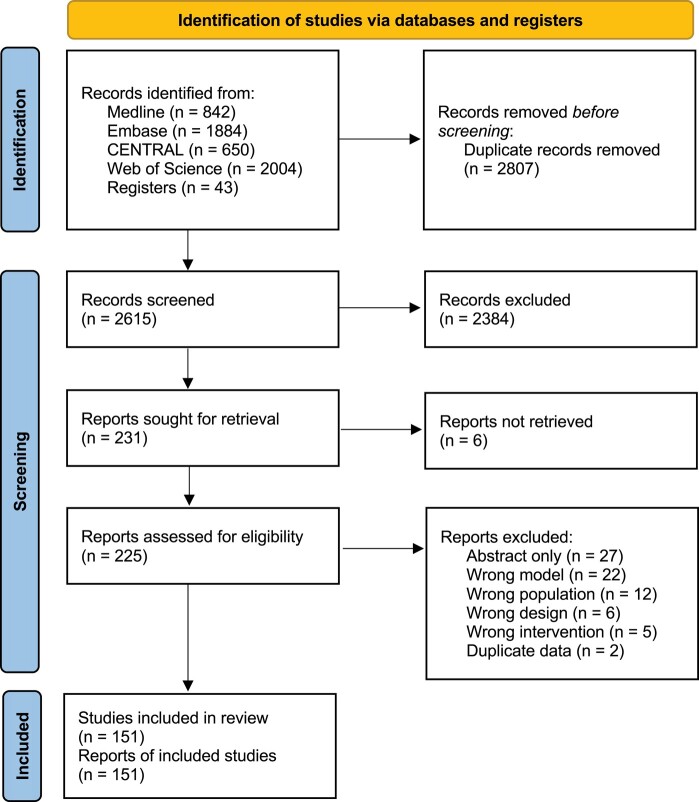
PRISMA flow diagram of study identification, screening and inclusion.

Our search strategy identified 5423 records, of which 151 population PK studies (120 prospective observational, 25 retrospective observational, 6 RCTs) with 6018 participants were included in the final review (Figure 1). The median [IQR (range)] number of participants recruited to each study was 24 [12–49 (6–285)]. We were not able to retrieve 6 reports, and identified 12 ongoing and 12 unpublished studies with unclear recruitment status. An overview of study characteristics grouped by study design is presented in Table 1. A complete list of studies is presented in Appendices S3–S5. A detailed table of study characteristics is presented in Table S1.

**Table 1.** Characteristics of included studies by study design

	Prospective observational, *n* (%) or median (IQR)	Retrospective observational, *n* (%) or median (IQR)	RCT, *n* (%) or median (IQR)	Total, *n* (%) or median (IQR)
Studies	120 (80)	25 (16)	6 (4)	151 (100)
Participants				
Total	3394 (56)	2455 (41)	169 (3)	6018 (100)
Sample size	24 (12–33)	70 (31–146)	19 (16–28)	24 (12–49)
Location				
Europe	59 (39)	13 (9)	4 (3)	76 (50)
Asia and Pacific	40 (27)	4 (3)	2 (1)	46 (30)
North America	17 (11)	6 (4)	—	23 (15)
International	4 (3)	1 (0.7)	—	5 (3)
Latin America & Caribbean	—	1 (0.7)	—	1 (0.7)
Income category				
High-income	107 (71)	23 (15)	6 (4)	136 (90)
Upper-middle-income	8 (5)	2 (1)	—	10 (7)
Lower-middle-income	5 (3)	—	—	5 (3)
Cohort/diagnosis				
Sepsis and septic shock	29 (19)	4 (3)	3 (2)	36 (24)
General ICU	25 (17)	9 (6)	—	34 (23)
Pneumonia	16 (11)	5 (3)	—	21 (14)
Burns	5 (3)	3 (2)	—	8 (5)
Trauma	5 (3)	1 (0.7)	—	6 (4)
Obesity	5 (3)	1 (0.7)	—	6 (4)
Extracorporeal support				
RRT	53 (35)	4 (3)	—	57 (38)
ECMO	14 (9)	1 (0.7)	—	15 (10)
Antimicrobials^a^				
Meropenem	43 (29)	9 (6)	1 (0.7)	53 (35)
Piperacillin	25 (16)	5 (3)	2 (1)	32 (21)
Cefepime	8 (5)	4 (3)	—	12 (8)
Doripenem	9 (6)	2 (1)	—	11 (7)
Imipenem	7 (5)	3 (2)	1 (0.7)	11 (7)
Ceftazidime	7 (5)	2 (1)	1 (0.7)	10 (7)
Ceftriaxone	7 (5)	1 (0.7)	—	8 (5)
Samples				
Plasma	96 (64)	18 (12)	5 (3)	119 (79)
Serum	21 (14)	7 (5)	1 (0.7)	29 (19)
Dialysis fluid	16 (11)	2 (1)	—	18 (12)
ELF	3 (2)	1 (0.7)	1 (0.7)	5 (3)
Urine	5 (3)	—	—	5 (3)
Subcutaneous tissue	4 (3)	—	1 (0.7)	5 (3)
CSF	3 (2)	—	—	3 (2)

ELF, epithelial lining fluid.

^a^Only antimicrobials used in more than five studies are shown here. For the complete description of antimicrobials used see the full list of included study characteristics in the Table S1.

**Figure 2.**
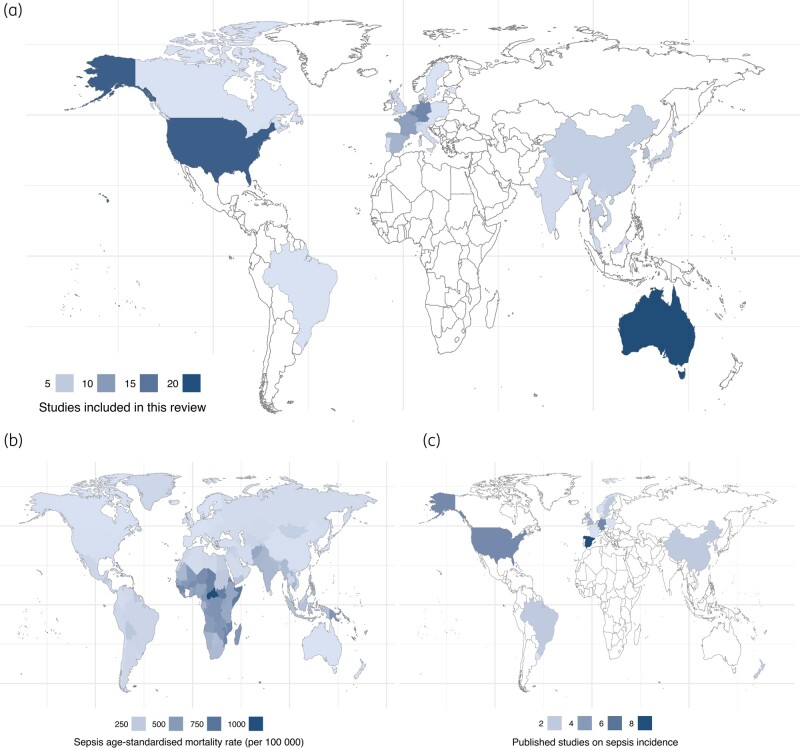
Global distribution of published population PK studies. Choropleth plots representing (a) publication location of studies included in this review, (b) sepsis age-standardized mortality rate according to the Global Burden of Disease study^2^ and (c) publication location of studies on sepsis incidence.^29^

The majority of included studies were conducted in high-income countries (90%), of which the most frequent were: Australia (23 studies, 15%); USA (22 studies, 15%); Belgium (14 studies, 10%); and Germany (15 studies, 10%). Only 16 (10%) studies were conducted in middle-income settings and none in low-income countries (Figure 2). In terms of participant cohorts and diagnoses, 36 (24%) studies looked at patients with sepsis and septic shock, 34 (22%) at general ICU patients, and 21 (14%) at patients with pneumonia. Extracorporeal support in the form of renal replacement therapy (RRT) and extracorporeal membrane oxygenation (ECMO) was used in 57 (38%) and 15 (10%) studies, respectively.

Five studies (3%) assessed more than one antimicrobial agent,^30–34^ whereas 34 (22%) of the included studies reported on combination agents, with piperacillin/tazobactam being the most commonly studied combination agent in 30 (20%) studies. We identified 66 (44%) studies looking at carbapenems, of which meropenem was the most commonly assessed antimicrobial in 53 (35%) studies. The most common primary samples were plasma in 119 (79%) and serum in 29 (19%) included studies. Eighteen (12%) studies reported quantification of antimicrobial concentration in some form of dialysis fluid (e.g. effluent or haemofiltrate). Other commonly sampled tissues were epithelial lining fluid (five studies, 3%),^35–39^ urine (five studies, 3%),^33,40–43^ subcutaneous tissue (five studies, 3%)^44–48^ and CSF (three studies, 2%).^49–51^ To quantify antimicrobial concentrations in blood, HPLC, either alone or in combination with LC-MS/MS, was used in 142 (93%) studies.

For the conduct of NLME modelling, NONMEM was the most commonly used software package (73 studies, 48%), followed by Pmetrics (40 studies, 26%) and Monolix (8 studies, 5%). Monte Carlo pharmacodynamic simulations were undertaken and reported in 115 (76%) studies. Models were assessed using visual predictive checks (90 studies, 59%), goodness-of-fit plots (80 studies, 53%), objective function values (57 studies, 38%), bootstrapping (50 studies, 33%) and the Akaike information criterion (48 studies, 32%). The method of covariate identification for testing was not reported in most of the identified studies (99 studies, 66%). When reported, covariates were chosen based on biological or clinical plausibility in 47 (31%) studies and selected from a range of previously published covariates in 6 (4%) studies. Expert consensus was used in one study. The covariate testing method was not explicitly reported in 35 (23%) studies, whereas the stepwise forward inclusion and backwards elimination method was reported to be used in 83 (55%) studies, followed by other methods such as the likelihood ratio test (21 studies, 14%), goodness-of-fit plots (13 studies, 9%) and Bayesian estimates (11 studies, 7%). A two-compartment model emerged as the best choice for fitting the studied antimicrobials in the majority of cases, with 110 studies (73%) supporting this finding.

### Reporting quality and risk of bias

Detailed reporting quality and risk-of-bias assessments are presented in Figures S3–S5).

### Reported covariates

**Figure 3.**
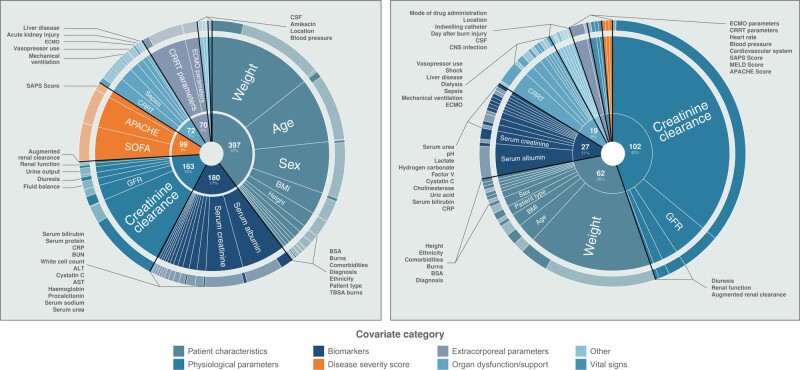
Sunburst plot of all published covariates with their respective significance and PK parameters. All covariates published in more than three studies are presented in (a) with covariate categories in the inner rim, covariates in the middle rim, and significance (shaded) and non-significance (light) denoted in the outer rim. Covariates reported as significant are presented in (b), where the outer rim represents the frequency of covariates tested on CL (shaded) or *V*_d_ (light), respectively.

We identified 1083 reported covariates, of which 237 were unique. Their frequencies are plotted in a sunburst diagram in Figure 3(a). Following categorization and thematic grouping we noted 120 unique covariates. Of all reported covariate instances, 240 (22%) were reported as significant. Covariates reported as significant in more than one study are summarized in Table 2, and those reported in more than one study are plotted along with their respective parameters in a sunburst diagram in Figure 3(b). Seven covariates were significant in more than 20% of the included studies, and were retained in the final models: CL_CR_; weight; glomerular filtration rate; diuresis; need for renal replacement; serum albumin; and C-reactive protein. The following covariates were reported as significant, only appearing in one study: cholinesterase; CNS infection; day after burn injury; dialysis (verbatim covariate used in one study for type of dialysis used); factor V; heart rate; hydrogen carbonate; indwelling catheter; lactate; Model for End-Stage Liver Disease score; mode of administration (of study drug); shock; and uric acid. A complete list of all verbatim reported covariates, including those reported in only one study, is presented in Appendix S6.

**Table 2.** Reported covariates per category

Covariate category	Covariate	Instances, number of studies	Significant, number of studies (%)
Patient characteristics		404	61 (15)
	Weight^a^	144	38 (26)
	Age	89	8 (9)
	Sex	71	3 (4)
	BMI	30	4 (13)
	Height	21	1 (5)
	BSA	10	1 (10)
	Burns	10	1 (10)
	Comorbidity	5	1 (20)
	Diagnosis	5	1 (20)
	Ethnicity	5	1 (20)
	Patient type	4	2 (50)
Biomarkers		206	31 (15)
	Serum albumin^a^	60	15 (25)
	Serum creatinine	51	4 (8)
	Serum bilirubin	12	2 (17)
	C-reactive protein^a^	9	2 (22)
	Cystatin C	4	1 (25)
	Serum urea	3	1 (33)
	pH	2	1 (50)
Physiological parameters		163	108 (66)
	CL_CR_^a^	108	86 (80)
	GFR^a^	21	12 (57)
	Diuresis^a^	8	5 (63)
	Renal function	5	3 (60)
	ARC	3	1 (33)
Disease severity score		103	4 (4)
	SOFA score	46	1 (2.1)
	APACHE score	43	1 (2.3)
	SAPS score	10	1 (10)
Organ dysfunction/support		83	18 (22)
	CRRT (requirement)^a^	20	7 (35)
	Sepsis	18	2 (11)
	Mechanical ventilation	12	2 (17)
	Vasopressor use	9	1 (11)
	ECMO (requirement)	7	3 (43)
	Liver disease	3	1 (33)
Extracorporeal parameters		74	10 (14)
	CRRT parameters	51	9 (18)
	ECMO parameters	19	1 (5)
Other		42	6 (14)
	CSF parameters	6	1 (17)
	Patient location	3	1 (33)
Vital signs		9	3 (33)
	Blood pressure	5	1 (20)

BSA, body surface area; CRRT, continuous renal replacement therapy; GFR, glomerular filtration rate.

^a^Covariates found to be significant more than 20% of the time reported in at least five studies.

We grouped reported covariates according to their categories and examined reported frequencies for each antimicrobial assessed in the included studies. Carbapenems were the most commonly studied antimicrobial category, with 589 (55%) covariates reported overall for this group of agents. This is graphically presented in heatmaps in Figures S6–S8).

## Discussion

Our review presented here is, to the best of our knowledge, the only systematic and comprehensive overview of published covariates used in population PK studies of critically ill adults receiving β-lactam antimicrobials. We identified 1083 covariates from 151 studies, with an overwhelming majority of studies being conducted in high-income countries; we identified no studies from low-income settings. In line with our original hypothesis, we demonstrated that only seven commonly investigated covariates (CL_CR_, weight, glomerular filtration rate, diuresis, requirement for renal replacement, serum albumin and C-reactive protein) are reported as significant in more than 20% of the included studies; of these, CL_CR_ is the most predictive of population clearance, in the respective study populations where it was explored.

### A framework for covariate selection

The main output of our work is an overview of covariates often chosen in population PK studies, and ultimately found to be significant and retained in NLME models, which we hope will help inform future consensus statements and societal recommendations on the exact covariates to choose when designing population PK studies. A small number of covariates in our review appeared to improve model fit overall. While we obviously cannot draw precise conclusions from such a heterogeneous pool of studies, it is perhaps worth considering these as a minimal essential dataset for future work, much like a core outcome set for clinical outcomes. Such work will build on current recommendations by Kanji *et al.*^27^ in any upcoming iterations or updates.

As part of this project, we created a GitHub repository of the dataset and associated scripts used using the *shiny* package in R; researchers can explore the dataset to select covariates tailored to their specific projects. The repository is accessible at https://ikb785-jan-hansel.shinyapps.io/popPKapp/ under the Creative Commons Attribution 4.0 International (CC BY 4.0) license.^52^ Future scope for expansion exists, as studies may be added to the dataset going forward, creating a living repository of individual patient-level data (e.g. measured antimicrobial concentrations for participants included in population PK studies). This would allow future quantitative synthesis, such as population PK meta-analyses, and the development of tailored models at reduced cost.^53^ As TDM of β-lactam antimicrobials becomes embedded into routine practice, access to samples and concentration measurements is foreseen to be more readily available, allowing straightforward pooling and modelling at scale. As a final output, the application can be expanded into a model-based precision dosing software solution.

### Novel covariates in critical illness: looking beyond the usual suspects

CL_CR_ clearance was a strong predictor of population clearance in the included studies; with nearly 80% of reports indicating a significant relationship and retention of CL_CR_ (either calculated or estimated) in the final proposed models, it is clear that the largest attributable contribution to model precision can be made through its inclusion. Interestingly, presumed or established ARC, defined as either estimated CL_CR_ (using the Cockcroft–Gault formula) > 130 mL/min or glomerular filtration rate (estimated using the CKD-EPI equation) > 96.5 mL/min/1.73 m^2^,^54^ although associated with suboptimal drug exposure, was reported in only five studies,^32,55–58^ and investigated as a covariate in two.^57,59^ One of these studies, looking at critically ill patients with pneumonia and preserved renal function, found the inclusion of ARC to improve model fit. This remains an under-investigated covariate.^60^

Biomarkers are frequently reported, but rarely found to be significant. This also holds true for illness severity scores; in our review, scores such as the APACHE score or SOFA score were very infrequently found to significantly influence model fit (2% studies). This is somewhat surprising, although it may be explained through the inclusion of these covariates into the structural model as a categorical rather than a continuous variable. In our dataset, inflammatory biomarkers (such as C-reactive protein, white cell count, procalcitonin, interleukin-6 and CD64 index) were only investigated 21 (2%) times in 10 studies.^35,61–69^ C-reactive protein was found to be significant in two of these studies, both studies of meropenem; one investigated septic patients on ECMO,^61^ while the other looked at a mixed medical/surgical ICU cohort with normal renal function.^66^ Of note, all of the above studies were published within the last 4 years, potentially indicating a trend towards increasing interest in the impact of inflammatory signatures on PK.

Among the rarely reported covariates, there was an eclectic mix of variables that were usually specific to the study population, and these are rarely found to be significant. However, it is not possible to exclude sizeable effects given the rarity of reporting of such covariates. Notably, our review highlights the distinct absence of investigations of potentially important patient characteristics, pertaining to their underlying disease process; specifically, there are no studies assessing the influence of sepsis subphenotypes on model fit. We therefore propose that future population PK research in septic patients should include identified subphenotypes as exploratory covariates.

### Population inequalities in population PK studies

Fewer than 10% of studies were conducted in settings that were not high income; of these, none were conducted in low-income settings. Our finding mirrors the established mismatch between the global disease burden of sepsis, and the magnitude of research outputs, where an inverse correlation can be observed based on country income groupings. Although access to TDM has been described as variable overall,^3^ central laboratories in LMICs are equipped with LC-MS/MS technology.^70^ Therefore, a hub-and-spoke model may be feasible even in these environments, although resource capacity and healthcare priorities remain important considerations.^70–72^

One recent population PK study included in our review looked at the blood samples of 24 Brazilian critically ill patients, specifically with a view to explore the interethnic variation within their cohort.^73^ Although a small population was studied and no piperacillin PK differences between ethnic groups for critically ill patients were observed, the authors noted that a dose adjustment based on CL_CR_ was required in Brazilian patients. It is not clear from the manuscript whether this adjustment is specific to their study cohort or whether this is an adjustment based on CL that would otherwise improve model fit in any population. Tsai *et al.*^74^ conducted a focused systematic review, identifying 50 studies that met their inclusion criteria, with six that specifically compared interethnic PK variability within the same study. While they noted considerable variations for certain types of antimicrobials and populations in general (e.g. individuals of Asian ethnicity achieved higher drug exposures than their Caucasian counterparts, likely to do with a comparatively lower *V*_d_ and CL), their work highlighted a knowledge gap with further work required to identify whether these findings are of clinical relevance to patient outcomes and other uncertainties. Our review highlights that this remains an under-investigated area.

Underdosing is an ongoing challenge strongly associated with the development of AMR, which continues to contribute to increased mortality, claiming up to 700 000 lives annually, and prolonged hospital admissions, particularly in LMICs.^75,76^ Factors that contribute to the development of AMR in LMICs, with examples relevant to population PK, can be classified as political, economic, sociological, technological, industry-related and ecological.^76^ Our review highlights ongoing disparities between high-income countries and LMICs. While these observations are subject to reporting bias, there is undoubtedly a skew in favour of high-income countries conducting (and reporting) research requiring specialized equipment and skilled staff; population pharmacokinetic studies are expensive and remain in the domain of well-resourced environments, as evidenced by our data.

### Limitations

Although a meta-regression of some of the findings from various models could have been undertaken, this was beyond the scope of this review’s aims and objectives. As anticipated, we discovered considerable heterogeneity in published population PK studies, limiting the usefulness of pooling quantitative parameters in a meaningful way. Combined with overall small study sample sizes, this is in fact one of the major limitations of the underlying data, and poses a methodological and practical challenge for future population PK work. With respect to reporting quality, Kanji and colleagues^27^ noted poorest compliance in items such as completeness of the abstract, fluid/tissue sampling, body weight metrics used, and descriptions of extracorporeal methods; by contrast, our review highlighted limited compliance with reporting of coadministered drugs, specific body weight, and study subject withdrawals being accounted for. We opted to use the Kanji ClinPK framework for quality assessment, but other tools are available, such as the Grading and Assessment of Pharmacokinetic-Pharmacodynamic Studies (GAPPS) system.^77^ This has, however, only been validated in paediatric populations. Limiting inclusion by location (only studies conducted in intensive care) may have excluded potentially eligible studies of patients who are cared for in poorly resourced environments with limited access to intensive care beds, but are in effect critically ill; this may have introduced a degree of selection bias. The covariate classification we derived was a bespoke categorization, and has not been externally validated; the coding may have therefore introduced bias.

### Conclusions

There are three main messages to take away from our review. First, our findings should help inform future covariate selection in population PK studies of β-lactam (and other) antimicrobials in the intensive care setting. We made the interactive list of covariates freely accessible (https://ikb785-jan-hansel.shinyapps.io/popPKapp/) to aid investigators in future study design. Second, it highlights the need to cast the net wider, and explore novel covariates that may inform future population PK models. A potential innovative avenue for investigation is the systematic testing of sepsis subphenotypes as categorical covariates in given populations. Third, the paucity of population PK studies conducted in LMICs is concerning, and warrants further study along with prioritized funding to characterize these at-risk populations. Models developed in high-income settings cannot simply be applied to other populations, therefore there is a need to develop tailored datasets from LMICs with the overall aim of decreasing global disparities in clinical outcomes of sepsis and septic shock.

## Supplementary Material

dlae030_Supplementary_Data

## Data Availability

The datasets generated as part of our work supporting the conclusions of this article are available in the GitHub repository (https://doi.org/10.5281/zenodo.8241522) and is available at https://github.com/jhanse/popPK_covariates.^52^

## Supplementary data

Figures S1 to S8, Table S1 and Appendices S1 to S6 are available as Supplementary data at *JAC-AMR* Online.
