# Supplementary material for: Covariates in population pharmacokinetic studies of critically ill adults receiving β-lactam antimicrobials: a systematic review and narrative synthesis
Source: JAC Antimicrob Resist. 2024 Feb 26;6(1):dlae030. doi: 10.1093/jacamr/dlae030 (PMC10895699; doi:10.1093/jacamr/dlae030)
Supplement: dlae030_Supplementary_Data [file dlae030_supplementary_data.docx]

**SUPPLEMENTARY DATA 1**

**Covariates in population pharmacokinetic studies of critically ill adults receiving beta-lactam antimicrobials: A systematic review and narrative synthesis**

Jan Hansel^1,2^, Fahmida Mannan^3^, Rebecca Robey^1^, Mary Kumarendran^2^, Siân Bladon^4^, Alexander Mathioudakis^1^, Kayode Ogungbenro^5^, Paul Dark^1^, Tim Felton^1,2^

^1^ Division of Immunology, Immunity to Infection and Respiratory Medicine, School of Biological Sciences, University of Manchester, Oxford Road, Manchester M13 9PL, UK

^2^ Acute Intensive Care Unit, Wythenshawe Hospital, Manchester University NHS Foundation Trust, Southmoor Road, Wythenshawe, Manchester M23 9LT, UK

^3^ Division of Cardiovascular Sciences, School of Medical Sciences, University of Manchester, Oxford Road, Manchester M13 9PL, UK

^4^ Division of Informatics, Imaging & Data Sciences, School of Health Sciences, University of Manchester, Oxford Road, Manchester M13 9PL, UK

^5^ Division of Pharmacy & Optometry, School of Health Sciences, University of Manchester, Oxford Road, Manchester M13 9PL, UK

**Corresponding Author:**

Jan Hansel

Division of Immunology, Immunity to Infection and Respiratory Medicine

School of Biological Sciences

University of Manchester

Oxford Road

+44 79608 19027

[Jan.hansel@nhs.net](mailto:Jan.hansel@nhs.net)

**Appendix S1: Search strategies**

Ovid MEDLINE(R) ALL <1946 to February 28, 2023>

| # | Query | Results from 1 Mar 2023 |
| --- | --- | --- |
| 1 | ((Concentration adj1 response) or (Exposure adj1 response) or ((Pharmacokinetic* or pharmaco-kinetic*) and (pharmacodynamic* or pharmaco-dynamic*)) or ("PK/PD" or "PK-PD") or ("pharmacokinetic*-pharmacodynamic*" or "pharmacokinetic*/pharmacodynamic*" or "pharmaco-kinetic*-pharmaco-dynamic*" or "pharmaco-kinetic*/pharmaco-dynamic*") or (Pharmacodynamic* ind* or Pharmaco-dynamic* ind* or PDI) or ("t>MIC" or "%t>MIC" or "ft>MIC" or "f%t>MIC" or "%fT>MIC" or "time>MIC" or "time above MIC") or ("C/MIC" or "Cmax/MIC" or "fC/MIC" or "fCmax/MIC" or "Cmin/MIC" or "fCmin/MIC" or "Cavg/MIC" or "fCavg/MIC" or "Peak/MIC" or "trough/MIC") or ("AUC*/MIC" or "fAUC*/MIC" or AUIC)).ti,ab. or Pharmacokinetics/ or pk.fs. or exp Area Under Curve/ or (AUC* or fAUC* or area under).ti,ab. or ((maximum or peak or trough or minimum or average) adj3 (concentration or level)).ti,ab. | 632677 |
| 2 | exp Lactams/ or (lactam* or beta-lactam* or streptogramin* or oxazolidinone* or lipopeptide*).ti,ab. or (Penicillin* or benzylpenicillin or penicillin G or phenoxymethylpenicillin or penicillin V or flucloxacillin or temocillin or ampicillin or amoxicillin or co-amoxiclav or amoxiclav or coamoxiclav or co-fluampicil or fluampicil or cofluampicil or piperacillin or ticarcillin or Mecillinam* or pivmecillinam).ti,ab. or (Ce*alosporin* or ce*aclor or ce*adroxil or ce*alexin or ce*ixime or ce*otaxime or ce*radine or ce*taroline fosamil or ce*tazidime or ce*triaxone or ce*uroxime).ti,ab. or (Carbapenem* or imipenem or meropenem or ertapenem).ti,ab. | 247657 |
| 3 | exp Critical Care/ or critical care.tw. or Critical Illness/ or critical$ ill$.tw. or exp Intensive Care Units/ or intensive care.tw. or (ICU$ or SICU$ or MICU$ or ITU$).tw. | 323529 |
| 4 | 1 and 2 and 3 | 1168 |
| 5 | exp animals/ not humans/ | 5098481 |
| 6 | 4 not 5 | 1155 |
| 7 | exp children/ not adults/ | 1422461 |
| 8 | 6 not 7 | 1106 |
| 9 | limit 8 to (autobiography or bibliography or biography or case reports or clinical conference or comment or congress or editorial or guideline or interview or lecture or letter or practice guideline or "review" or "scientific integrity review" or video-audio media or webcast) | 264 |
| 10 | 8 not 9 | 842 |

Embase <1974 to 2023 February 28>

| # | Query | Results from 1 Mar 2023 |
| --- | --- | --- |
| 1 | ((Concentration adj1 response) or (Exposure adj1 response) or ((Pharmacokinetic* or pharmaco-kinetic*) and (pharmacodynamic* or pharmaco-dynamic*)) or ("PK/PD" or "PK-PD") or ("pharmacokinetic*-pharmacodynamic*" or "pharmacokinetic*/pharmacodynamic*" or "pharmaco-kinetic*-pharmaco-dynamic*" or "pharmaco-kinetic*/pharmaco-dynamic*") or (Pharmacodynamic* ind* or Pharmaco-dynamic* ind* or PDI) or ("t>MIC" or "%t>MIC" or "ft>MIC" or "f%t>MIC" or "%fT>MIC" or "time>MIC" or "time above MIC") or ("C/MIC" or "Cmax/MIC" or "fC/MIC" or "fCmax/MIC" or "Cmin/MIC" or "fCmin/MIC" or "Cavg/MIC" or "fCavg/MIC" or "Peak/MIC" or "trough/MIC") or ("AUC*/MIC" or "fAUC*/MIC" or AUIC)).ti,ab. or Pharmacokinetics/ or pk.fs. or exp Area Under Curve/ or (AUC* or fAUC* or area under).ti,ab. or ((maximum or peak or trough or minimum or average) adj3 (concentration or level)).ti,ab. | 1115858 |
| 2 | exp Lactams/ or (lactam* or beta-lactam* or streptogramin* or oxazolidinone* or lipopeptide*).ti,ab. or (Penicillin* or benzylpenicillin or penicillin G or phenoxymethylpenicillin or penicillin V or flucloxacillin or temocillin or ampicillin or amoxicillin or co-amoxiclav or amoxiclav or coamoxiclav or co-fluampicil or fluampicil or cofluampicil or piperacillin or ticarcillin or Mecillinam* or pivmecillinam).ti,ab. or (Ce*alosporin* or ce*aclor or ce*adroxil or ce*alexin or ce*ixime or ce*otaxime or ce*radine or ce*taroline fosamil or ce*tazidime or ce*triaxone or ce*uroxime).ti,ab. or (Carbapenem* or imipenem or meropenem or ertapenem).ti,ab. | 611751 |
| 3 | exp Critical Care/ or critical care.tw. or Critical Illness/ or critical$ ill$.tw. or exp Intensive Care Units/ or intensive care.tw. or (ICU$ or SICU$ or MICU$ or ITU$).tw. | 1147368 |
| 4 | 1 and 2 and 3 | 3835 |
| 5 | exp animals/ not humans/ | 11644566 |
| 6 | 4 not 5 | 2826 |
| 7 | exp children/ not adults/ | 2319318 |
| 8 | 6 not 7 | 2532 |
| 9 | limit 8 to (autobiography or bibliography or biography or case reports or clinical conference or comment or congress or editorial or guideline or interview or lecture or letter or practice guideline or "review" or "scientific integrity review" or video-audio media or webcast) [Limit not valid in Embase; records were retained] | 648 |
| 10 | 8 not 9 | 1884 |

Cochrane CENTRAL <to 28 February 2023>

| # | Query | Results from 1 Mar 2023 |
| --- | --- | --- |
| 1 | population pharmacokinetics OR population pharmacokinetic OR pharmacokinetic OR pharmacokinetics OR pharmacodynamic OR pharmacodynamics OR AUC OR MIC OR concentration OR level OR peak OR maximum OR trough | |
| 2 | critical care OR intensive care OR ICU OR ITU OR critical OR intensive OR critically ill | |
| 3 | lactams OR lactam OR beta-lactam OR beta lactam OR penicillin OR flucloxacillin OR temocillin OR ampicillin OR amoxicillin OR co-amoxiclav OR piperacillin OR ticarcillin OR mecillinam OR cephalosporin OR cefalosporin OR cefalexine OR cephalexine OR cefuroxime OR cefotaxime OR ceftazidime OR ceftoraline OR meropenem OR ertapenem OR imipenem OR carbapenem OR doripenem | |
| 4 | #1 AND #2 AND #3 | 650 |

Web of Science <to 28 February 2023>

| # | Query | Results from 1 Mar 2023 |
| --- | --- | --- |
| 1 | #1 ts=(concentration NEAR/1 response) OR ts=(exposure NEAR/1 response) OR ts=((Pharmacokinetic* OR pharmaco-kinetic*) AND (pharmacodynamic* or pharmaco-dynamic*)) OR ts=("PK/PD" OR "PK-PD") OR ts=("pharmacokinetic*-pharmacodynamic*" OR "pharmacokinetic*/pharmacodynamic*" OR "pharmaco-kinetic*-pharmaco-dynamic*" OR "pharmaco-kinetic*/pharmaco-dynamic*") OR ts=(Pharmacodynamic ind* or PDI) OR ts=("*t>MIC" OR "time>MIC" OR "time above MIC") OR ts=("C/MIC" OR "Cmax/MIC" OR "fC/MIC" OR "fCmax/MIC" OR "Cmin/MIC" OR "fCmin/MIC" OR "Cavg/MIC" OR "fCavg/MIC" OR "Peak/MIC" OR "trough/MIC" OR "*C*/MIC") OR ts=("*AUC*/MIC" or AUIC) | |
| 2 | #2 ts=critical* ill* OR ts=(Critical Care) OR ts=(critical NEAR/1 care) OR ts=(Critical Illness) OR ts=critical* OR ts=(Intensive Care Unit*) OR ts=(ICU or SICU or MICU or ITU) | |
| 3 | #3 ts=("anti-microbial" OR antimicrobial OR antibacter* OR anti-bacter* OR antibiotic* OR anti-biotic* OR *lactam* OR *Penicillin* OR *cillin* OR *ocillin* OR *amoxiclav OR *fluampicil OR *mecillinam* OR Ce*alosporin* OR ce*aclor OR ce*adroxil OR ce*alexin OR ce*ixime OR ce*otaxime OR ce*radine OR ce*taroline OR ce*tazidime OR ce*triaxone OR ce*uroxime OR *penem*) | |
| 4 | #1 AND #2 AND #3 | 2004 |

**ClinicalTrials.gov <25 June 2023>**

| # | Query | Results from 25 June 2023 |
| --- | --- | --- |
| 1 | (population pharmacokinetics OR population pharmacokinetic) AND (critical care OR intensive care OR ICU OR ITU) AND (lactams OR lactam OR beta-lactam OR beta lactam OR flucloxacillin OR temocillin OR ampicillin OR amoxicillin OR co-amoxiclav OR piperacillin OR ticarcillin OR mecillinam OR cephalosporin OR cefalosporin OR cefalexine OR cephalexine OR cefuroxime OR cefotaxime OR ceftazidime OR ceftoraline OR meropenem OR ertapenem OR imipenem OR carbapenem) | 43 |

**Appendix S2: Data extraction form**

| **Study characteristics extraction form template** | | | | | | |
| --- | --- | --- | --- | --- | --- | --- |
|  |  |  |  |  |  |  |
| **doi** |  | **extractor1** |  | **extractor2** |  | **study_id** |
| DOI link |  | Extractor 1 initials |  | Extractor 2 initials |  | Fixed study ID |
|  |  |  |  |  |  |  |
| **author** |  | **year** |  | **country** |  | **study_design** |
| Study author surname |  | Publication year |  | Country |  | Study design |
|  |  |  |  |  |  |  |
| **number_participants** |  | **inclusion_criteria** |  | **exclusion_criteria** |  | **extracorporeal** |
| Number of participants |  | Inclusion criteria |  | Exclusion criteria |  | Extracorporeal support |
|  |  |  |  |  |  |  |
| **pt_characteristics** |  | **other_pt_groups** |  | **samples_used** |  | **other_samples** |
| Special populations |  | Other non-critically ill patients studied |  | Primary tissue sample used |  | Non-blood tissue samples used |
|  |  |  |  |  |  |  |
| **antimicrobial** |  | **other_antimicrobials** |  | **quantification_method** |  | **modelling_software** |
| Beta-lactam of primary interest |  | Other reported antimicrobial(s) |  | Quantification method(s) |  | Modelling software used |
|  |  |  |  |  |  |  |
| **compartments** |  | **model_evaluation** |  | **covariate_identification** |  | **covar_testing_method** |
| Number of compartments |  | Methods used for model evaluation |  | Method(s) of covariate identification |  | Method(s) of covariate testing |
|  |  |  |  |  |  |  |
| **method_for_PTA** |  | **covariates_reported** |  | **comments** |  |  |
| Monte Carlo simulations used |  | Dichotomous yes/no |  | Other comments |  |  |
|  |  |  |  |  |  |  |
|  |  |  |  |  |  |  |
| **Covariate extraction form template** | | | | | | |
|  |  |  |  |  |  |  |
| **study_id** |  | **covariate** |  | **covariate_category** |  | **significant** |
| Fixed study ID |  | Verbatim reported covariate |  | Covariate category assignment |  | Covariate reported as significant |
|  |  |  |  |  |  |  |
| **covariate_parameter** |  | **comments** |  |  |  |  |
| Parameter for which covariate significant |  | Other comments |  |  |  |  |

**Appendix S3: References to included studies**

1. Abdul-Aziz, M. H., et al. (2016). ‘Population Pharmacokinetics of Doripenem in Critically Ill Patients with Sepsis in a Malaysian Intensive Care Unit’, *Antimicrobial Agents & Chemotherapy*, 60(1), pp. 206-14. doi:10.1128/AAC.01543-15.
2. Al-Shaer, M. H., et al. (2022). ‘Applying Cefepime Population Pharmacokinetics to Critically Ill Patients Receiving Continuous Renal Replacement Therapy’, *Antimicrobial Agents & Chemotherapy*, 66(1), p. 18. doi:10.1128/AAC.01611-21.
3. Al-Shaer, M. H., et al. (2020). ‘Population Pharmacokinetics and Target Attainment of Cefepime in Critically Ill Patients and Guidance for Initial Dosing’, *Antimicrobial Agents & Chemotherapy*, 64(9), p. 20. doi:10.1128/AAC.00745-20.
4. Al-Shaer, M. H., et al. (2021). ‘Cefepime Population Pharmacokinetics and Target Attainment in Critically Ill Patients on Continuous Renal Replacement Therapy’, *Antimicrobial Agents & Chemotherapy*, 65(6), p. 18. doi:10.1128/AAC.00144-21.
5. Alobaid, A. S., et al. (2017). ‘Population Pharmacokinetics of Piperacillin in Nonobese, Obese, and Morbidly Obese Critically Ill Patients’, *Antimicrobial Agents & Chemotherapy*, 61(3), p. 03. doi:10.1128/AAC.01276-16.
6. Alobaid, A. S., et al. (2016). ‘Effect of Obesity on the Population Pharmacokinetics of Meropenem in Critically Ill Patients’, *Antimicrobial Agents & Chemotherapy*, 60(8), pp. 4577-84. doi:10.1128/AAC.00531-16.
7. An, G., et al. (2023). ‘Evaluation of Empirical Dosing Regimens for Meropenem in Intensive Care Unit Patients Using Population Pharmacokinetic Modeling and Target Attainment Analysis’, *Antimicrobial Agents & Chemotherapy*, 67(1), p. 24. doi:10.1128/aac.01312-22.
8. Asin-Prieto, E., et al. (2014). ‘Population pharmacokinetics of piperacillin and tazobactam in critically ill patients undergoing continuous renal replacement therapy: application to pharmacokinetic/pharmacodynamic analysis’, *Journal of Antimicrobial Chemotherapy*, 69(1), pp. 180-9. doi:10.1093/jac/dkt304.
9. Barreto, E. F., et al. (2023). ‘Adequacy of cefepime concentrations in the early phase of critical illness: A case for precision pharmacotherapy’, *Pharmacotherapy.* doi:10.1002/phar.2766.
10. Bastida, C., et al. (2020). ‘Meropenem population pharmacokinetics in patients with decompensated cirrhosis and severe infections’, *Journal of Antimicrobial Chemotherapy*, 75(12), pp. 3619-3624. doi:10.1093/jac/dkaa362.
11. Benitez-Cano, A., et al. (2020). ‘Intrapulmonary concentrations of meropenem administered by continuous infusion in critically ill patients with nosocomial pneumonia: a randomized pharmacokinetic trial’, *Critical Care*, 24(1), p. 17. doi:10.1186/s13054-020-2763-4.
12. Bhalodi, A. A., et al. (2013). ‘Pharmacokinetics of doripenem in infected patients treated within and outside the intensive care unit’, *Annals of Pharmacotherapy*, 47(5), pp. 617-27. doi:10.1345/aph.1R789.
13. Boonpeng, A., et al. (2022). ‘Population Pharmacokinetics/Pharmacodynamics and Clinical Outcomes of Meropenem in Critically Ill Patients’, *Antimicrobial Agents & Chemotherapy*, 66(11), p. 15. doi:10.1128/aac.00845-22.
14. Braune, S., et al. (2018). ‘Pharmacokinetics of meropenem in septic patients on sustained low-efficiency dialysis: a population pharmacokinetic study’, *Critical Care*, 22(1), p. 30. doi:10.1186/s13054-018-1940-1.
15. Bue, M., et al. (2020). ‘Population pharmacokinetics of piperacillin in plasma and subcutaneous tissue in patients on continuous renal replacement therapy’, *International Journal of Infectious Diseases*, 92, pp. 133-140. doi:10.1016/j.ijid.2020.01.010.
16. Buning, A. W., et al. (2021). ‘Population pharmacokinetics and probability of target attainment of different dosing regimens of ceftazidime in critically ill patients with a proven or suspected pseudomonas aeruginosa infection’, *Antibiotics*, 10(6), p. 612. doi:10.3390/antibiotics10060612.
17. Burger, R., et al. (2018). ‘Effect of renal clearance and continuous renal replacement therapy on appropriateness of recommended meropenem dosing regimens in critically ill patients with susceptible life-threatening infections’, *Journal of Antimicrobial Chemotherapy*, 73(12), pp. 3413-3422. doi:10.1093/jac/dky370.
18. Burkhardt, O., et al. (2007). ‘Ertapenem in critically ill patients with early-onset ventilator-associated pneumonia: Pharmacokinetics with special consideration of free-drug concentration’, *Journal of Antimicrobial Chemotherapy*, 59(2), pp. 277-284. doi:10.1093/jac/dkl485.
19. Carlier, M., et al. (2013). ‘Population pharmacokinetics and dosing simulations of amoxicillin/clavulanic acid in critically ill patients’, *Journal of Antimicrobial Chemotherapy*, 68(11), pp. 2600-8. doi:10.1093/jac/dkt240.
20. Carlier, M., et al. (2014). ‘Population pharmacokinetics and dosing simulations of cefuroxime in critically ill patients: non-standard dosing approaches are required to achieve therapeutic exposures’, *Journal of Antimicrobial Chemotherapy*, 69(10), pp. 2797-803. doi:10.1093/jac/dku195.
21. Chauzy, A., et al. (2022). ‘Population pharmacokinetic/pharmacodynamic study suggests continuous infusion of ceftaroline daily dose in ventilated critical care patients with early-onset pneumonia and augmented renal clearance’, *The Journal of antimicrobial chemotherapy*, 77(11), pp. 3173-3179. doi:10.1093/jac/dkac299.
22. Chauzy, A., et al. (2019). ‘Cerebrospinal fluid pharmacokinetics of ceftaroline in neurosurgical patients with an external ventricular drain’, *Journal of Antimicrobial Chemotherapy*, 74(3), pp. 675-681. doi:10.1093/jac/dky489.
23. Cheatham, S. C., et al. (2014). ‘Steady-state pharmacokinetics and pharmacodynamics of meropenem in morbidly obese patients hospitalized in an intensive care unit’, *Journal of Clinical Pharmacology*, 54(3), pp. 324-30. doi:10.1002/jcph.196.
24. Chen, W., et al. (2020). ‘Imipenem Population Pharmacokinetics: Therapeutic Drug Monitoring Data Collected in Critically Ill Patients with or without Extracorporeal Membrane Oxygenation’, *Antimicrobial Agents & Chemotherapy*, 64(6), p. 21. doi:10.1128/AAC.00385-20.
25. Cheng, V., et al. (2021). ‘Population Pharmacokinetics of Piperacillin and Tazobactam in Critically Ill Patients Receiving Extracorporeal Membrane Oxygenation: an ASAP ECMO Study’, *Antimicrobial Agents & Chemotherapy*, 65(11), p. 18. doi:10.1128/AAC.01438-21.
26. Cheng, V., et al. (2022). ‘Population Pharmacokinetics and Dosing Simulations of Ceftriaxone in Critically Ill Patients Receiving Extracorporeal Membrane Oxygenation (An ASAP ECMO Study)’, *Clinical Pharmacokinetics*, 61(6), pp. 847-856. doi:10.1007/s40262-021-01106-x.
27. Cheng, V., et al. (2021). ‘Population pharmacokinetics of cefepime in critically ill patients receiving extracorporeal membrane oxygenation (an ASAP ECMO study)’, *International Journal of Antimicrobial Agents*, 58(6). doi:10.1016/j.ijantimicag.2021.106466.
28. Chung, E. K., et al. (2017). ‘Population Pharmacokinetics and Pharmacodynamics of Doripenem in Obese, Hospitalized Patients’, *Annals of Pharmacotherapy*, 51(3), pp. 209-218. doi:10.1177/1060028016676831.
29. Cojutti, P. G., et al. (2021). ‘Impact of Maximizing Css/MIC Ratio on Efficacy of Continuous Infusion Meropenem Against Documented Gram-Negative Infections in Critically Ill Patients and Population Pharmacokinetic/Pharmacodynamic Analysis to Support Treatment Optimization’, *Frontiers in Pharmacology*, 12, p. 781892. doi:10.3389/fphar.2021.781892.
30. Conil, J. M., et al. (2007). ‘A population pharmacokinetic approach to ceftazidime use in burn patients: Influence of glomerular filtration, gender and mechanical ventilation’, *British Journal of Clinical Pharmacology*, 64(1), pp. 27-35. doi:10.1111/j.1365-2125.2007.02857.x.
31. Couffignal, C., et al. (2014). ‘Population pharmacokinetics of imipenem in critically ill patients with suspected ventilator-associated pneumonia and evaluation of dosage regimens’, *British Journal of Clinical Pharmacology*, 78(5), pp. 1022-34. doi:10.1111/bcp.12435.
32. Crandon, J. L., et al. (2011). ‘Optimization of meropenem dosage in the critically ill population based on renal function’, *Intensive Care Medicine*, 37(4), pp. 632-8. doi:10.1007/s00134-010-2105-0.
33. De Jongh, R., et al. (2008). ‘Continuous versus intermittent infusion of temocillin, a directed spectrum penicillin for intensive care patients with nosocomial pneumonia: Stability, compatibility, population pharmacokinetic studies and breakpoint selection’, *Journal of Antimicrobial Chemotherapy*, 61(2), pp. 382-388. doi:10.1093/jac/dkm467.
34. de Velde, F., et al. (2020). ‘Population Pharmacokinetics of Imipenem in Critically Ill Patients: A Parametric and Nonparametric Model Converge on CKD-EPI Estimated Glomerular Filtration Rate as an Impactful Covariate’, *Clinical Pharmacokinetics*, 59(7), pp. 885-898. doi:10.1007/s40262-020-00859-1.
35. Delattre, I. K., et al. (2012). ‘Population pharmacokinetics of four beta-lactams in critically ill septic patients comedicated with amikacin’, *Clinical Biochemistry*, 45(10), pp. 780-6. doi:10.1016/j.clinbiochem.2012.03.030.
36. Dhaese, S. A. M., et al. (2019). ‘Saturable elimination of piperacillin in critically ill patients: implications for continuous infusion’, *International Journal of Antimicrobial Agents*, 54(6), pp. 741-749. doi:10.1016/j.ijantimicag.2019.08.024.
37. Dhaese, S. A. M., et al. (2019). ‘Population pharmacokinetics and evaluation of the predictive performance of pharmacokinetic models in critically ill patients receiving continuous infusion meropenem: a comparison of eight pharmacokinetic models’, *Journal of Antimicrobial Chemotherapy*, 74(2), pp. 432-441. doi:10.1093/jac/dky434.
38. Dhaese, S. A. M., et al. (2018). ‘Population pharmacokinetics of continuous infusion of piperacillin in critically ill patients’, *International Journal of Antimicrobial Agents*, 51(4), pp. 594-600. doi:10.1016/j.ijantimicag.2017.12.015.
39. Dinh, T. D., et al. (2022). ‘Population-Based Pharmacokinetics and Dose Optimization of Imipenem in Vietnamese Critically-Ill Patients’, *Infection and Drug Resistance*, 15, pp. 4575-4583. doi:10.2147/IDR.S373348.
40. Dreesen, E., et al. (2022). ‘Ceftriaxone dosing based on the predicted probability of augmented renal clearance in critically ill patients with pneumonia’, *Journal of Antimicrobial Chemotherapy*, 77(9), pp. 2479-2488. doi:10.1093/jac/dkac209.
41. Economou, C. J. P., et al. (2019). ‘Population pharmacokinetics of ticarcillin in critically ill patients receiving extended daily diafiltration’, *International Journal of Antimicrobial Agents*, 54(3), pp. 351-355. doi:10.1016/j.ijantimicag.2019.06.027.
42. Ehmann, L., et al. (2019). ‘Development of a dosing algorithm for meropenem in critically ill patients based on a population pharmacokinetic/pharmacodynamic analysis’, *International Journal of Antimicrobial Agents*, 54(3), pp. 309-317. doi:10.1016/j.ijantimicag.2019.06.016.
43. Eisert, A., et al. (2021). ‘Comparison of two empirical prolonged infusion dosing regimens for meropenem in patients with septic shock: A two-center pilot study’, *International Journal of Antimicrobial Agents*, 57(3). doi:10.1016/j.ijantimicag.2021.106289.
44. Eyler, R. F., et al. (2014). ‘Pharmacokinetics of ertapenem in critically ill patients receiving continuous venovenous hemodialysis or hemodiafiltration’, *Antimicrobial Agents & Chemotherapy*, 58(3), pp. 1320-6. doi:10.1128/AAC.02090-12.
45. Felton, T. W., et al. (2014). ‘Pulmonary penetration of piperacillin and tazobactam in critically ill patients’, *Clinical Pharmacology & Therapeutics*, 96(4), pp. 438-48. doi:10.1038/clpt.2014.131.
46. Felton, T. W., et al. (2018). ‘Comparison of piperacillin exposure in the lungs of critically ill patients and healthy volunteers’, *Journal of Antimicrobial Chemotherapy*, 73(5), pp. 1340-1347. doi:10.1093/jac/dkx541.
47. Felton, T. W., et al. (2014). ‘Individualization of piperacillin dosing for critically ill patients: dosing software to optimize antimicrobial therapy’, *Antimicrobial Agents & Chemotherapy*, 58(7), pp. 4094-102. doi:10.1128/AAC.02664-14.
48. Fillatre, P., et al. (2021). ‘Impact of extracorporeal membrane oxygenation (ECMO) support on piperacillin exposure in septic patients: a case-control study’, *The Journal of Antimicrobial Chemotherapy*, 76(5), pp. 1242-9. doi:10.1093/jac/dkab031.
49. Fournier, A., et al. (2018). ‘Population Pharmacokinetic Study of Amoxicillin-Treated Burn Patients Hospitalized at a Swiss Tertiary-Care Center’, *Antimicrobial Agents & Chemotherapy*, 62(9), p. 09. doi:10.1128/AAC.00505-18.
50. Fratoni, A. J., et al. (2022). ‘Imipenem/cilastatin/relebactam pharmacokinetics in critically ill patients with augmented renal clearance’, *The Journal of Antimicrobial Chemotherapy.*, 77(11), pp. 2992-9. doi:10.1093/jac/dkac261.
51. Frippiat, F., et al. (2015). ‘Modelled target attainment after meropenem infusion in patients with severe nosocomial pneumonia: the PROMESSE study’, *Journal of Antimicrobial Chemotherapy*, 70(1), pp. 207-16. doi:10.1093/jac/dku354.
52. Garot, D., et al. (2011). ‘Population pharmacokinetics of ceftriaxone in critically ill septic patients: a reappraisal’, *British Journal of Clinical Pharmacology*, 72(5), pp. 758-67. doi:10.1111/j.1365-2125.2011.04005.x.
53. Georges, B., et al. (2009). ‘Population pharmacokinetics of ceftazidime in intensive care unit patients: influence of glomerular filtration rate, mechanical ventilation, and reason for admission’, *Antimicrobial Agents & Chemotherapy*, 53(10), pp. 4483-9. doi:10.1128/AAC.00430-09.
54. Gijsen, M., et al. (2022). ‘Meropenem Target Attainment and Population Pharmacokinetics in Critically Ill Septic Patients with Preserved or Increased Renal Function’, *Infection & Drug Resistance*, 15, pp. 53-62. doi:10.2147/IDR.S343264.
55. Goncalves-Pereira, J., et al. (2014). ‘Assessment of pharmacokinetic changes of meropenem during therapy in septic critically ill patients’, *BMC Pharmacology & Toxicology*, 15(21), p. 14. doi:10.1186/2050-6511-15-21.
56. Grensemann, J., et al. (2020). ‘Acute-on-chronic liver failure alters meropenem pharmacokinetics in critically ill patients with continuous hemodialysis: an observational study’, *Annals of Intensive Care*, 10, p. 48. doi:10.1186/s13613-020-00666-8.
57. Hahn, J., et al. (2021). ‘Population Pharmacokinetics and Dosing Optimization of Piperacillin-Tazobactam in Critically Ill Patients on Extracorporeal Membrane Oxygenation and the Influence of Concomitant Renal Replacement Therapy’, *Microbiology Spectrum*, 9(3), p. 22. doi:10.1128/Spectrum.00633-21.
58. Hanberg, P., et al. (2018). ‘Population Pharmacokinetics of Meropenem in Plasma and Subcutis from Patients on Extracorporeal Membrane Oxygenation Treatment’, *Antimicrobial Agents & Chemotherapy*, 62(5), p. 05. doi:10.1128/AAC.02390-17.
59. Heffernan, A. J., et al. (2022). ‘Multicenter Population Pharmacokinetic Study of Unbound Ceftriaxone in Critically Ill Patients’, *Antimicrobial Agents & Chemotherapy*, 66(6), p. 21. doi:10.1128/aac.02189-21.
60. Idoate Grijalba, A. I., et al. (2019). ‘Evaluation of a non-parametric modelling for meropenem in critically ill patients using Monte Carlo simulation’, *European Journal of Clinical Pharmacology*, 75(10), pp. 1405-1414. doi:10.1007/s00228-019-02716-y.
61. Isla, A., et al. (2008). ‘Population pharmacokinetics of meropenem in critically ill patients undergoing continuous renal replacement therapy’, *Clinical Pharmacokinetics*, 47, pp. 173-180. doi:10.2165/00003088-200847030-00003.
62. Jacobs, A., et al. (2018). ‘beta-Lactam Dosage Regimens in Septic Patients with Augmented Renal Clearance’, *Antimicrobial Agents & Chemotherapy*, 62(9), p. 09. doi:10.1128/AAC.02534-17.
63. Jager, N. G. L., et al. (2020). ‘Optimization of flucloxacillin dosing regimens in critically ill patients using population pharmacokinetic modelling of total and unbound concentrations’, *Journal of Antimicrobial Chemotherapy*, 75(9), pp. 2641-2649. doi:10.1093/jac/dkaa187.
64. Jaruratanasirikul, S., et al. (2015). ‘Population pharmacokinetics and Monte Carlo dosing simulations of meropenem during the early phase of severe sepsis and septic shock in critically ill patients in intensive care units’, *Antimicrobial Agents & Chemotherapy*, 59(6), pp. 2995-3001. doi:10.1128/AAC.04166-14.
65. Jaruratanasirikul, S., et al. (2019). ‘Pharmacokinetics of Imipenem in Critically Ill Patients with Life-threatening Severe Infections During Support with Extracorporeal Membrane Oxygenation’, *Clinical Drug Investigation*, 39(8), pp. 787-798. doi:10.1007/s40261-019-00796-3.
66. Jeon, S., et al. (2014). ‘Population pharmacokinetic analysis of piperacillin in burn patients’, *Antimicrobial Agents & Chemotherapy*, 58(7), pp. 3744-51. doi: 10.1128/AAC.02089-13.
67. Jonckheere, S., et al. (2016). ‘A model-based analysis of the predictive performance of different renal function markers for cefepime clearance in the ICU’, *Journal of Antimicrobial Chemotherapy*, 71(9), pp. 2538-2546. doi:10.1093/jac/dkw171.
68. Kang, S., et al. (2022). ‘Dose Optimization of Meropenem in Patients on Veno-Arterial Extracorporeal Membrane Oxygenation in Critically Ill Cardiac Patients: Pharmacokinetic/Pharmacodynamic Modeling’, *Journal of Clinical Medicine*, 11(22), p. 08. doi:10.3390/jcm11226621.
69. Kanji, S., et al. (2018). ‘Piperacillin Population Pharmacokinetics in Critically Ill Adults During Sustained Low-Efficiency Dialysis’, *Annals of Pharmacotherapy*, 52(10), pp. 965-973. doi:10.1177/1060028018773771.
70. Kees, M. G., et al. (2016). ‘Population pharmacokinetics of meropenem during continuous infusion in surgical ICU patients’, *Journal of Clinical Pharmacology*, 56(3), pp. 307-15. doi:10.1002/jcph.600.
71. Kim, Y. K., et al. (2022). ‘Population pharmacokinetics of piperacillin/tazobactam in critically ill Korean patients and the effects of extracorporeal membrane oxygenation’, *Journal of Antimicrobial Chemotherapy*, 77(5), pp. 1353-1364. doi:10.1093/jac/dkac059.
72. Klastrup, V., et al. (2020). ‘Population Pharmacokinetics of Piperacillin following Continuous Infusion in Critically Ill Patients and Impact of Renal Function on Target Attainment’, *Antimicrobial Agents & Chemotherapy*, 64(7), p. 23. doi:10.1128/AAC.02556-19.
73. Kois, A. K., et al. (2022). ‘Pharmacokinetics and Time above the MIC Exposure of Cefepime in Critically Ill Patients Receiving Extracorporeal Membrane Oxygenation (ECMO)’, *International Journal of Antimicrobial Agents*, 60(1). doi:10.1016/j.ijantimicag.2022.106603.
74. Konig, C., et al. (2017). ‘Population pharmacokinetics and dosing simulations of ceftazidime in critically ill patients receiving sustained low-efficiency dialysis’, *Journal of Antimicrobial Chemotherapy*, 72(5), pp. 1433-40. doi:10.1093/jac/dkw592.
75. Kothekar, A. T., et al. (2020). ‘Clinical pharmacokinetics of 3-h extended infusion of meropenem in adult patients with severe sepsis and septic shock: implications for empirical therapy against Gram-negative bacteria’, *Annals of Intensive Care*, 10(1): p. 04. doi:10.1186/s13613-019-0622-8.
76. Krueger, W. A., et al. (1998). ‘Pharmacokinetics of meropenem in critically ill patients with acute renal failure treated by continuous hemodiafiltration’, *Antimicrobial Agents and Chemotherapy*, 42(9), pp. 2421-4. doi:10.1128/aac.42.9.2421.
77. Kumta, N., et al. (2022). ‘Plasma and Cerebrospinal Fluid Population Pharmacokinetics of Meropenem in Neurocritical Care Patients: a Prospective Two-Center Study’, *Antimicrobial Agents & Chemotherapy*, 66(8), p. 16. doi:10.1128/aac.00142-22.
78. Layios, N., et al. (2022). ‘Modelled Target Attainment after Temocillin Treatment in Severe Pneumonia: Systemic and Epithelial Lining Fluid Pharmacokinetics of Continuous versus Intermittent Infusions’, *Antimicrobial Agents & Chemotherapy*, 66(3), p. 15. doi:10.1128/AAC.02052-21.
79. Lee, D. H., et al. (2021). ‘Population Pharmacokinetics of Meropenem in Critically Ill Korean Patients and Effects of Extracorporeal Membrane Oxygenation’, *Pharmaceutics*, 13(11), p. 04. doi:10.3390/pharmaceutics13111861.
80. Lee, J. H., et al. (2021). ‘Pharmacokinetics and Monte Carlo Simulation of Meropenem in Critically Ill Adult Patients Receiving Extracorporeal Membrane Oxygenation’, *Frontiers in Pharmacology*, 12, p. 768912. doi:10.3389/fphar.2021.768912.
81. Leegwater, E., et al. (2020). ‘Population pharmacokinetics of ceftriaxone administered as continuous or intermittent infusion in critically ill patients’, *The Journal of Antimicrobial Chemotherapy.*, 75(6), pp. 1554-8. doi:10.1093/jac/dkaa067.
82. Li, S. and Xie, F. (2019). ‘Population pharmacokinetics and simulations of imipenem in critically ill patients undergoing continuous renal replacement therapy’, *International Journal of Antimicrobial Agents*, 53(1), pp. 98-105. doi:10.1016/j.ijantimicag.2018.10.006.
83. Li, Z., et al. (2020). ‘Pharmacokinetic and Pharmacodynamic Analysis of Critically Ill Patients Undergoing Continuous Renal Replacement Therapy With Imipenem’, *Clinical therapeutics.*, 42(8), pp. 1564-1577.e8. doi:10.1016/j.clinthera.2020.06.010.
84. Liebchen, U., et al. (2021). ‘Evaluation of the MeroRisk Calculator, A User-Friendly Tool to Predict the Risk of Meropenem Target Non-Attainment in Critically Ill Patients’, *Antibiotics*, 10(4), p. 20. doi:10.3390/antibiotics10040468.
85. Lipman, J., Wallis, S. C. and Rickard, C. (1999). ‘Low plasma cefepime levels in critically ill septic patients: Pharmacokinetic modeling indicates improved troughs with revised dosing’, *Antimicrobial Agents and Chemotherapy*, 43(10), pp. 2559-61. doi:10.1128/aac.43.10.2559.
86. Lipman, J., et al. (2001). ‘Low cefpirome levels during twice daily dosing in critically ill septic patients: Pharmacokinetic modelling calls for more frequent dosing’, *Intensive Care Medicine*, 27(2), pp. 363-70. doi:10.1007/s001340000741.
87. Mathew, S. K., et al. (2016). ‘A Nonparametric Pharmacokinetic Approach to Determine the Optimal Dosing Regimen for 30-Minute and 3-Hour Meropenem Infusions in Critically Ill Patients’, *Therapeutic Drug Monitoring*, 38(5), pp. 593-9. doi:10.1097/FTD.0000000000000323.
88. Mattioli, F., et al. (2016). ‘Population pharmacokinetics and probability of target attainment of meropenem in critically ill patients’, *European Journal of Clinical Pharmacology*, 72(7), pp. 839-48. doi:10.1007/s00228-016-2053-x.
89. Minichmayr, I. K., et al. (2018). ‘Development of a dosing nomogram for continuous-infusion meropenem in critically ill patients based on a validated population pharmacokinetic model’, *Journal of Antimicrobial Chemotherapy*, 73(5), pp. 1330-9. doi:10.1093/jac/dkx526.
90. Mueller, S. C., et al. (2002). ‘Pharmacokinetics of piperacillin-tazobactam in anuric intensive care patients during continuous venovenous hemodialysis’, *Antimicrobial Agents and Chemotherapy*, 46(5), pp. 1557-60. doi:10.1128/AAC.46.5.1557-1560.2002.
91. Murinova, I., et al. (2022). ‘Meropenem population pharmacokinetics and model-based dosing optimisation in patients with serious bacterial infection’, *European Journal of Hospital Pharmacy*. doi:10.1136/ejhpharm-2022-003535.
92. Nandy, P., Samtani, M. N. and Lin, R. (2010). ‘Population pharmacokinetics of doripenem based on data from phase 1 studies with healthy volunteers and phase 2 and 3 studies with critically ill patients’, *Antimicrobial Agents & Chemotherapy*, 54(6), pp. 2354-9. doi:10.1128/AAC.01649-09.
93. Nicasio, A. M., et al. (2009). ‘Population pharmacokinetics of high-dose, prolonged-infusion cefepime in adult critically ill patients with ventilator-associated pneumonia’, *Antimicrobial Agents & Chemotherapy*, 53(4), pp. 1476-81. doi:10.1128/AAC.01141-08.
94. Niibe, Y., et al. (2020). ‘Population Pharmacokinetic Analysis of Meropenem in Critically Ill Patients With Acute Kidney Injury Treated With Continuous Hemodiafiltration’, *Therapeutic Drug Monitoring*, 42(4), pp. 588-594. doi:10.1097/FTD.0000000000000741.
95. Niibe, Y., et al. (2022). ‘Identification of factors affecting meropenem pharmacokinetics in critically ill patients: Impact of inflammation on clearance’, *Journal of Infection and Chemotherapy*, 28(4), pp. 532-8. doi:10.1016/j.jiac.2021.12.017.
96. Nonoshita, K., et al. (2020). ‘Population pharmacokinetic analysis of doripenem for Japanese patients in intensive care unit’, *Scientific Reports*, 10(1), p. 17. doi:10.1038/s41598-020-79076-6.
97. O'Jeanson, A., et al. (2021). ‘Population Pharmacokinetics and Pharmacodynamics of Meropenem in Critically Ill Patients: How to Achieve Best Dosage Regimen According to the Clinical Situation’, *European Journal of Drug Metabolism & Pharmacokinetics*, 46(5), pp. 695-705. doi: 10.1007/s13318-021-00709-w.
98. Öbrink-Hansen, K., et al. (2015). ‘Population pharmacokinetics of piperacillin in the early phase of septic shock: does standard dosing result in therapeutic plasma concentrations?’, *Antimicrob Agents Chemother*, 59(11), pp. 7018-26. doi: 10.1128/aac.01347-15.
99. Ollivier, J., et al. (2019). ‘Are standard dosing regimens of ceftriaxone adapted for critically ill patients with augmented creatinine clearance?’, *Antimicrobial Agents and Chemotherapy*, 63(3), pp. e02134-18. doi: 10.1128/AAC.02134-18.
100. Onichimowski, D., et al. (2020). ‘Population pharmacokinetics of standard-dose meropenem in critically ill patients on continuous renal replacement therapy: a prospective observational trial’, *Pharmacological Reports: PR*, 72(3), pp. 719-729. doi:10.1007/s43440-020-00104-3.
101. Padulles Zamora, A., et al. (2019). ‘Optimized meropenem dosage regimens using a pharmacokinetic/pharmacodynamic population approach in patients undergoing continuous venovenous haemodiafiltration with high-adsorbent membrane’, *Journal of Antimicrobial Chemotherapy*, 74(10), pp. 2979-2983. doi:10.1093/jac/dkz299.
102. Pokem, P. N., et al. (2022). ‘Population Pharmacokinetics of Temocillin Administered by Continuous Infusion in Patients with Septic Shock Associated with Intra-Abdominal Infection and Ascitic Fluid Effusion’, *Antibiotics*, 11(7), p. 898. doi:10.3390/antibiotics11070898.
103. Por, E. D., et al. (2021). ‘Population Pharmacokinetic Modeling and Simulations of Imipenem in burn Patients With and Without Continuous Venovenous Hemofiltration in the Military Health System’, *Journal of Clinical Pharmacology*, 61(9), pp. 1182-94. doi:10.1002/jcph.1865.
104. Rahbar, A. J., et al. (2016). ‘Pharmacokinetic and Pharmacodynamic Evaluation of Doripenem in Critically Ill Trauma Patients with Sepsis’, *Surgical Infections*, 17(6), pp. 675-682. doi:10.1089/sur.2015.113.
105. Robatel, C., et al. (2003). ‘Pharmacokinetics and Dosage Adaptation of Meropenem during Continuous Venovenous Hemodiafiltration in Critically Ill Patients’, *Journal of Clinical Pharmacology*, 43(12), pp. 1329-40. doi:10.1177/0091270003260286.
106. Roberts, D. M., et al. (2015). ‘A multicenter study on the effect of continuous hemodiafiltration intensity on antibiotic pharmacokinetics’, *Critical Care*, 19(84), p. 13. doi:10.1186/s13054-015-0818-8.
107. Roberts, J. A., et al. (2010). ‘First-dose and steady-state population pharmacokinetics and pharmacodynamics of piperacillin by continuous or intermittent dosing in critically ill patients with sepsis’, *International Journal of Antimicrobial Agents*, 35(2), pp. 156-63. doi:10.1016/j.ijantimicag.2009.10.008.
108. Roberts, J. A., et al. (2009). ‘Meropenem dosing in critically ill patients with sepsis and without renal dysfunction: intermittent bolus versus continuous administration? Monte Carlo dosing simulations and subcutaneous tissue distribution’, *Journal of Antimicrobial Chemotherapy*, 64(1), pp. 142-50. doi:10.1093/jac/dkp139.
109. Roberts, J. A. and Lipman, J. (2013). ‘Optimal doripenem dosing simulations in critically ill nosocomial pneumonia patients with obesity, augmented renal clearance, and decreased bacterial susceptibility’, *Critical Care Medicine*, 41(2), pp. 489-95. doi:10.1097/CCM.0b013e31826ab4c4.
110. Roberts, J. A., et al. (2009). ‘Piperacillin penetration into tissue of critically ill patients with sepsis--bolus versus continuous administration?’, *Critical Care Medicine*, 37(3), pp. 926-33. doi:10.1097/CCM.0b013e3181968e44.
111. Roberts, J. A., et al. (2014). ‘Doripenem population pharmacokinetics and dosing requirements for critically ill patients receiving continuous venovenous haemodiafiltration’, *Journal of Antimicrobial Chemotherapy*, 69(9), pp. 2508-16. doi:10.1093/jac/dku177.
112. Roberts, J. A., et al. (2015). ‘Plasma and target-site subcutaneous tissue population pharmacokinetics and dosing simulations of cefazolin in post-trauma critically ill patients’, *Journal of Antimicrobial Chemotherapy*, 70(5), pp. 1495-502. doi:10.1093/jac/dku564.
113. Roelofsen, E. E., et al. (2023). ‘Dose optimization of cefotaxime as pre-emptive treatment in critically ill adult patients: A population pharmacokinetic study’, *British Journal of Clinical Pharmacology*, 89(2), pp. 705-13. doi:10.1111/bcp.15487.
114. Rohani, R., et al. (2022). ‘Individual target pharmacokinetic/pharmacodynamic attainment rates among meropenem-treated patients admitted to the ICU with hospital-acquired pneumonia’, *The Journal of antimicrobial chemotherapy*, 77(11), pp. 2956-9. doi:10.1093/jac/dkac245.
115. Roos, J. F., et al. (2006). ‘Pharmacokinetic-pharmacodynamic rationale for cefepime dosing regimens in intensive care units’, *The Journal of antimicrobial chemotherapy*, 58(5), pp. 987-93. doi:10.1093/jac/dkl349.
116. Roos, J. F., Lipman, J. and Kirkpatrick, C. M. J. (2007). ‘Population pharmacokinetics and pharmacodynamics of cefpirome in critically ill patients against Gram-negative bacteria’, *Intensive Care Medicine*, 33(5), pp. 781-788. doi:10.1007/s00134-007-0573-7.
117. Sakka, S. G., et al. (2007). ‘Population pharmacokinetics and pharmacodynamics of continuous versus short-term infusion of imipenem-cilastatin in critically ill patients in a randomized, controlled trial’, *Antimicrobial Agents and Chemotherapy*, 51(9), pp. 3304-10. doi:10.1128/AAC.01318-06.
118. Sanches, C., et al. (2022). ‘Population Pharmacokinetic Model of Piperacillin in Critically Ill Patients and Describing Interethnic Variation Using External Validation’, *Antibiotics*, 11(4), p. 434. doi:10.3390/antibiotics11040434.
119. Selig, D. J., et al. (2022). ‘Comparison of Piperacillin and Tazobactam Pharmacokinetics in Critically Ill Patients with Trauma or with Burn’, *Antibiotics*, 11(5), p. 04. doi:10.3390/antibiotics11050618.
120. Selig, D. J., et al. (2022). ‘Meropenem pharmacokinetics in critically ill patients with or without burn treated with or without continuous veno-venous haemofiltration’, *British Journal of Clinical Pharmacology*, 88(5), pp. 2156-68. doi:10.1111/bcp.15138.
121. Shekar, K., et al. (2014). ‘The combined effects of extracorporeal membrane oxygenation and renal replacement therapy on meropenem pharmacokinetics: a matched cohort study’, *Critical Care*, 18(6), p. 12. doi:10.1186/s13054-014-0565-2.
122. Shotwell, M. S., et al. (2016). ‘Pharmacokinetics and pharmacodynamics of extended infusion versus short infusion piperacillin-tazobactam in critically Ill patients undergoing CRRT’, *Clinical journal of the American Society of Nephrology*, 11(8), pp. 1377‐1383. doi:10.2215/CJN.10260915.
123. Sime, F. B., et al. (2019). ‘Population Pharmacokinetics of Unbound Ceftolozane and Tazobactam in Critically Ill Patients without Renal Dysfunction’, *Antimicrobial Agents & Chemotherapy*, 63(10), p. 10. doi:10.1128/AAC.01265-19.
124. Sime, F. B., et al. (2020). ‘A population pharmacokinetic model-guided evaluation of ceftolozane-tazobactam dosing in critically ill patients undergoing continuous venovenous hemodiafiltration’, *Antimicrobial Agents and Chemotherapy*, 64(1), pp. e01655-19. doi:10.1128/AAC.01655-19.
125. Sime, F. B., et al. (2021). ‘Cerebrospinal fluid penetration of ceftolozane-tazobactam in critically ill patients with an indwelling external ventricular drain’, *Antimicrobial Agents and Chemotherapy*, 65(1), pp. e01698-20. doi:10.1128/AAC.01698-20.
126. Stein, G. E., et al. (2019). ‘Pharmacokinetic and Pharmacodynamic Analysis of Ceftazidime/Avibactam in Critically Ill Patients’, *Surgical Infections*, 20(1), pp. 55-61. doi:10.1089/sur.2018.141.
127. Sturm, A. W., et al. (2014). ‘Pharmacokinetic analysis of piperacillin administered with tazobactam in critically ill, morbidly obese surgical patients’, *Pharmacotherapy:The Journal of Human Pharmacology & Drug Therapy*, 34(1), pp. 28-35. doi:10.1002/phar.1324.
128. Sukarnjanaset, W., Jaruratanasirikul, S. and Wattanavijitkul, T. (2019). ‘Population pharmacokinetics and pharmacodynamics of piperacillin in critically ill patients during the early phase of sepsis’, *Journal of Pharmacokinetics & Pharmacodynamics*, 46(3), pp. 251-261. doi:10.1007/s10928-019-09633-8.
129. Swartling, M., et al. (2022). ‘Population pharmacokinetics of cefotaxime in intensive care patients’, *European Journal of Clinical Pharmacology*, 78(2), pp. 251-258. doi:10.1007/s00228-021-03218-6.
130. Tamme, K., et al. (2015). ‘Pharmacokinetics of doripenem during high volume hemodiafiltration in patients with septic shock’, *Journal of Clinical Pharmacology*, 55(4), pp. 438-46. doi:10.1002/jcph.432.
131. Tegeder, I., et al. (1997). ‘Pharmacokinetics of imipenem-cilastatin in critically ill patients undergoing continuous venovenous hemofiltration’, *Antimicrobial Agents and Chemotherapy*, 41(12), pp. 2640-5. doi:10.1128/aac.41.12.2640.
132. Tegeder, I., et al. (1999). ‘Pharmacokinetics of meropenem in critically ill patients with acute renal failure undergoing continuous venovenous hemofiltration’, *Clinical Pharmacology and Therapeutics*, 65(1), pp. 50-57. doi:10.1016/S0009-9236(99)70121-9.
133. Thuy, N. T. T., et al. (2018). ‘Population pharmacokinetics of meropenem in Vietnamese adult patients’, *Pharmaceutical Sciences Asia*, 45(4), pp. 221-30. doi:10.29090/psa.2018.04.017.0042.
134. Truong, A. Q., et al. (2022). ‘Optimizing Meropenem in Highly Resistant Klebsiella pneumoniae Environments: Population Pharmacokinetics and Dosing Simulations in Critically Ill Patients’, *Antimicrobial Agents and Chemotherapy*, 66(11). doi:10.1128/aac.00321-22.
135. Tsai, D., et al. (2016). ‘Optimising meropenem dosing in critically ill Australian Indigenous patients with severe sepsis’, *International Journal of Antimicrobial Agents*, 48(5), pp. 542-546. doi:10.1016/j.ijantimicag.2016.08.015.
136. Tsai, D., et al. (2016). ‘Pharmacokinetics of Piperacillin in Critically Ill Australian Indigenous Patients with Severe Sepsis’, *Antimicrobial Agents & Chemotherapy*, 60(12), pp. 7402-6. doi:10.1128/AAC.01657-16.
137. Udy, A. A., et al. (2015). ‘Are standard doses of piperacillin sufficient for critically ill patients with augmented creatinine clearance?’, *Critical Care*, 19(28), p. 30. doi:10.1186/s13054-015-0750-y.
138. Ulldemolins, M., et al. (2021). ‘Once-daily 1 g ceftriaxone optimizes exposure in patients with septic shock and hypoalbuminemia receiving continuous veno-venous hemodiafiltration’, *European Journal of Clinical Pharmacology*, 77(8), pp. 1169-80. doi:10.1007/s00228-021-03100-5.
139. Ulldemolins, M., et al. (2016). ‘Piperacillin population pharmacokinetics in critically ill patients with multiple organ dysfunction syndrome receiving continuous venovenous haemodiafiltration: effect of type of dialysis membrane on dosing requirements’, *Journal of Antimicrobial Chemotherapy*, 71(6), pp. 1651-9. doi:10.1093/jac/dkv503.
140. Ulldemolins, M., et al. (2010). ‘Flucloxacillin dosing in critically ill patients with hypoalbuminaemia: special emphasis on unbound pharmacokinetics’, *Journal of Antimicrobial Chemotherapy*, 65(8), pp. 1771-8. doi:10.1093/jac/dkq184.
141. Ulldemolins, M., et al. (2015). ‘Meropenem population pharmacokinetics in critically ill patients with septic shock and continuous renal replacement therapy: influence of residual diuresis on dose requirements’, *Antimicrobial Agents & Chemotherapy*, 59(9), pp. 5520-8. doi:10.1128/AAC.00712-15.
142. Van Dalen, R., et al. (1986). ‘Dosage adjustment for ceftazidime in patients with impaired renal function’, *European Journal of Clinical Pharmacology*, 30(5), pp. 597-605. doi:10.1007/BF00542421.
143. Van Dalen, R., Vree, T. B. and Baars, I. M. (1987). ‘Influence of protein binding and severity of illness on renal elimination of four cephalosporin drugs in intensive-care patients’, *Pharmaceutisch Weekblad - Scientific Edition*, 9(2), pp. 98-103. doi:10.1007/BF01960743.
144. van der Werf, T. S., et al. (1997). ‘Pharmacokinetics of piperacillin and tazobactam in critically ill patients with renal failure, treated with continuous veno-venous hemofiltration (CVVH)’, *Intensive Care Medicine*, 23(8), pp. 873-7. doi:10.1007/s001340050424.
145. Vossen, M. G., et al. (2015). ‘Doripenem Treatment during Continuous Renal Replacement Therapy’, *Antimicrobial Agents & Chemotherapy*, 60(3), pp. 1687-94. doi:10.1128/AAC.01801-15.
146. Wallenburg, E., et al. (2021). ‘High unbound flucloxacillin fraction in critically ill patients’, *The Journal of antimicrobial chemotherapy*, 76(12), pp. 3220-8. doi:10.1093/jac/dkab314.
147. Wallenburg, E., et al. (2022). ‘An Integral Pharmacokinetic Analysis of Piperacillin and Tazobactam in Plasma and Urine in Critically Ill Patients’, *Clinical Pharmacokinetics*, 61(6), pp. 907-918. doi:10.1007/s40262-022-01113-6.
148. Westermann, I., et al. (2021). ‘Population pharmacokinetics and probability of target attainment in patients with sepsis under renal replacement therapy receiving continuous infusion of meropenem: sustained low-efficiency dialysis (SLED) and continuous veno-venous haemodialysis (CVVHD)’, *British journal of clinical pharmacology*, 87(11), pp. 4293-303. doi:10.1111/bcp.14846.
149. Young, R. J., et al. (1997). ‘Intermittent bolus dosing of ceftazidime in critically ill patients’, *Journal of Antimicrobial Chemotherapy*, 40(2), pp. 269-73. doi:10.1093/jac/40.2.269.
150. Zahr, N., et al. (2022). ‘Total and Unbound Pharmacokinetics of Cefiderocol in Critically Ill Patients’, *Pharmaceutics*, 14(12), p. 2786. doi:10.3390/pharmaceutics14122786.
151. Zhao, Y. C., et al. (2022). ‘Does Prolonged Infusion Time Really Improve the Efficacy of Meropenem Therapy? A Prospective Study in Critically Ill Patients’, *Infectious Diseases & Therapy*, 11(1), pp. 201-16. doi:10.1007/s40121-021-00551-2.

**Appendix S4: References to studies not retrieved**

1. Georges, B., et al. (2008). ‘Cefepime in intensive care unit patients: Validation of a population pharmacokinetic approach and influence of covariables’, *International Journal of Clinical Pharmacology and Therapeutics*, 46(4), pp. 157-64. doi:10.5414/CPP46157.
2. Kihara, M., et al. (1994). ‘Pharmacokinetic profiles of intravenous imipenem/cilastatin during slow hemodialysis in critically ill patients’, *Clinical Nephrology*, 42(3), pp. 193-7.
3. Rondanelli, R., et al. (1986). ‘Ceftazidime in the treatment of Pseudomonas infections in intensive-care patients’, *International Journal of Clinical Pharmacology, Therapy, & Toxicology*, 24(9), pp. 457-9.
4. Meenks, S. D., et al. (2022). ‘Population pharmacokinetics of unbound ceftriaxone in a critically ill population’, *International Journal of Clinical Pharmacology & Therapeutics*, 60(9), pp. 373-83. doi:10.5414/CP204181.
5. Chen, W.Q. (2020). ‘A Comparative Study on Predicting %T>MIC Imipenem based on Therapeutic Drug Monitoring Results in Critically Care Patients’, *Chinese Pharmaceutical Journal*, 24, pp. 755-60.
6. Chaijamorn, W., et al. (2019). ‘Carbapenem dosing recommendations in critically ill patients receiving continuous renal replacement therapy’, Nephrology, 23. doi:10.1111/nep.13651

**Appendix S5: References to ongoing studies**

**Ongoing studies**

1. NCT05807217. (2022). A Pharmacokinetics Study of Meropenem and Piperacillin in Patients With Sepsis. https://classic.clinicaltrials.gov/show/NCT05807217.
2. NCT04033029. (2021). Antibiotic Plasma Concentrations During Continuous Renal Replacement Therapy With a High Adsorption Membrane (oXiris®). https://classic.clinicaltrials.gov/show/NCT04033029.
3. NCT05681442. (2023). Beta-lactam Intermittent Versus Continuous Infusion and Combination Antibiotic Therapy in Sepsis. https://classic.clinicaltrials.gov/show/NCT05681442.
4. NCT05862402. (2023). Dose Optimization by Pharmacokinetic/Pharmacodynamic of Antibiotics to Improve Clinical Outcome of Carbapenem Resistant Klebsiella Pneumoniae Bloodstream Infections in Critically Ill Patients at Phramongkutklao Hospital. https://classic.clinicaltrials.gov/show/NCT05862402.
5. NCT05146154. (2023). Impact of Obesity on the Pharmacokinetics of Imipenem-Relebactam in ICU Patients. https://classic.clinicaltrials.gov/show/NCT05146154.
6. NCT05566665. (2023). Nosocomial Infections in Patients With ARDS Treated With ECMO. https://classic.clinicaltrials.gov/show/NCT05566665.
7. NCT03990467. (2021). Observed Pharmacokinetic of Piperacillin/Tazobactam Compared to Amikacin in ICU. https://classic.clinicaltrials.gov/show/NCT03990467.
8. NCT05134298. (2019). Pharmacokinetics of Piperacillin and Meropenem in ICU Patients. https://classic.clinicaltrials.gov/show/NCT05134298.
9. NCT03748095. (2019). Population Pharmacokinetic Modeling to Optimize the Dosage of the Piperacillin Tazobactam Combination in Patients With Sepsis in Intensive Care. https://classic.clinicaltrials.gov/show/NCT03748095.
10. NCT04799626. (2021). Population Pharmacokinetics and Dosage Individualization of Antibiotics in Elderly Patients. https://classic.clinicaltrials.gov/show/NCT04799626.
11. NCT05024565. (2021). Prolonged Intravenous Infusion of β-lactam Antibiotics in Early Septic Patients. https://classic.clinicaltrials.gov/show/NCT05024565.
12. Vincze, I., et al. (2022). ‘Assessment of Antibiotic Pharmacokinetics, Molecular Biomarkers and Clinical Status in Critically Ill Adults Diagnosed with Community-Acquired Pneumonia and Receiving Intravenous Piperacillin/Tazobactam and Hydrocortisone over the First Five Days of Intensive Care: An Observational Study (STROBE Compliant)’, *Journal of Clinical Medicine*, 11(14), p. 4140.

**Unpublished studies with unclear recruitment status**

1. NCT02609646. (2016). AbioKin - Antibiotic Kinetics. https://classic.clinicaltrials.gov/show/NCT02609646.
2. NCT03915236. (2019). Approach for Optimizing Meropenem Therapy in Intubated and Mechanically-Ventilated, Adult Patients With Severe Gram-Negative Lower Respiratory Tract Infection. https://classic.clinicaltrials.gov/show/NCT03915236.
3. NCT01198925. (2010). Assessment of the Optimal Dosing of Piperacillin-tazobactam in Intensive Care Unit Patients: Extended Versus Continuous Infusion. https://classic.clinicaltrials.gov/show/NCT01198925.
4. NCT03404089. (2018). Bedside Therapeutic Monitoring of β-Lactam Levels in Newborns, Children and Adolescents Admitted to Intensive Care. https://classic.clinicaltrials.gov/show/NCT03404089.
5. NCT02917486. (2016). EC-MOTION : ExtraCorporeal Membrane Oxygenation and Therapeutic Drug Monitoring of Drugs of infectION. https://classic.clinicaltrials.gov/show/NCT02917486.
6. NCT01796717. (2012). Optimizing Dosing Regimen of Piperacillin/Tazobactam for Nosocomial Pneumonia. https://classic.clinicaltrials.gov/show/NCT01796717.
7. NCT02670239. (2014). Pharmacokinetics of Imipenem During Ex Vivo Lung Perfusion (EVLP). https://classic.clinicaltrials.gov/show/NCT02670239.
8. NCT02478073. (2015). Piperacillin Pharmacokinetics in ICU Patients. https://classic.clinicaltrials.gov/show/NCT02478073.
9. NCT03787550. (2020). PK/PD of the Sedatives, Analgesics and Antibiotics in Patients Receiving ECMO. https://classic.clinicaltrials.gov/show/NCT03787550.
10. NCT03481569. (2018). Population Pharmacokinetic-pharmacodynamic (PK-PD) Study of 9 Broad-Spectrum Anti-infective Agents in the Cerebro Spinal Fluid (CSF) of Brain Injured Patients With an External Ventricular Drainage (EVD). https://classic.clinicaltrials.gov/show/NCT03481569.
11. NCT03339869. (2018). Therapeutic Drug Monitoring of Anti-infectious Drugs in Intensive Care Unit. https://classic.clinicaltrials.gov/show/NCT03339869.
12. NCT05426499. (2021). Translational PKPD Modeling of Anti-infective Drugs Used in Pediatric Units. https://classic.clinicaltrials.gov/show/NCT05426499.

**Appendix S6: Comma-separated list of raw unique covariates reported**

abbreviated burn severity index, actual body weight, acute and chronic kidney disease, acute kidney injury, acute-on-chronic liver failure, adjusted body weight, age, alanine aminotransferase, alanine transaminase, albumin binding, albumin concentration, albumin, alcohol consumption, alkaline phosphatase, alkaline reserve, amikacin clearance, amikacin concentration measured 1 h after the start of infusion, amikacin concentration measured 8 h after start of infusion, amikacin V1, aminotransferase, antithrombin, APACHE II score, APACHE IV score, APACHE score, APAHCE II score, ARDS, ascitic fluid albumin, aspartate aminotransferase, aspartate transaminase, augmented renal clearance, bacteraemia, Baux index, bilirubin, blood flow rate, blood pressure, blood urea nitrogen, body mass index, body size, body surface area, body temperature, body weight gain, body weight loss, body weight, Body weight, BUN, burns, cardiovascular system, CD64 index, Charlson comorbidity index, CHDF hemofilter, CHDF intensity, cholinesterase, cirrhosis, comorbidities, comorbidity, concomitant medications used, concomitant medications, course of treatment, creatinine clearance, CRP, CRRT 6h prior to urine output, CRRT blood flow, CRRT dialysate flow, CRRT downtime, CRRT duration, CRRT effluent rate, CRRT intensity, CRRT membrane type, CRRT mode, CRRT type, CRRT ultrafiltrate net flow, CRRT, CSF compartment, CSF protein concentration, CSF protein:serum albumin ratio, CVVH, CVVHDF settings, cystatin C, daily fluid intake, day after burn injury, day of antibiotic therapy, day of filter usage, days in ICU, degree of diuresis, dehydration, dialysate flow rate, dialysate plus ultrafiltrate flow, dialysate slow rate, dialysis, diastolic blood pressure, diuresis, diuretic use, drug sieving coefficient, ECMO duration, ECMO flow rate, ECMO mode, ECMO pump speed, ECMO termination, ECMO type, ECMO, effluent rate, effluent volume, ethnicity, factor V, fibrinogen, filter downtime, filter type, fluid balance, fluid flow rate, fluid intake, fluid output, fluid overload, fluid removal volume, furosemide use, gamma glutamyl transferase, glomerular filtration rate, haematocrit, haemoglobin, health status, heart rate, height, history of liver disease, hydrogencarbonate, ICU, ideal body weight, indwelling catheter, infectious diagnosis, infusion volume, interleukin-6, KIM-1, lactate dehydrogenase, lactate, lean body mass, lean body weight, liver failure, location, lung transplantation, mean arterial pressure, measured creatinine clearance, mechanical ventilation, MELD score, membrane type, mode of administration, MODS score, mortality, noradrenaline use, occasion number, oedema score, oedema, other comorbidities, P/F ratio, patient population, patient type, pCO2, PCT, PEEP, percentage of burned total body surface area, peritonitis, pH, plasma albumin, platelets, postoperative central nervous system infection, predicted probability of ARC, Presence of CSF infection, primary diagnosis, procalcitonin, pulse oximetry, race, re-admission, reason for admission, red cell count, renal function, replacement fluid flow rate, repository flow rate, residual diuresis volume, residual diuresis, RIFLE score, rotations per minute, RRT, SAPS 1 score, SAPS 2 score, SAPS 3 score, sepsis severity, sepsis, septic shock, serum alanine aminotransferase, serum albumin, serum bilirubin, serum creatinine, serum protein, serum sodium, serum urea, severe sepsis, sex, shock, site of infection, SLED, smoking status, SOFA score, study phase, study site, surgical drain, systolic blood pressure, targeted hourly fluid removal, therapy fluid flow rate, time on RRT, time since ECMO initiation, Tobiasen index, total amount of resuscitation fluids daily, total body surface area, total body weight change, total body weight, total burn surface area, total effluent flow rate, total second degree burn surface area, total second-degree burn surface area, total serum protein, total third degree burn surface area, total third-degree burn surface area, transaminase, transmembrane pressure, treatment, type of anticoagulation, ultrafiltrate flow rate, unbound drug fraction, unbound fraction of drug, unit, urea, uric acid, urine output, uromodulin, vasoactive medications, vasopressor use, volume of CSF collected from ventricular drain, volume of CSF drained over sampling interval, weight, white cell count

| Table S1: Characteristics of included studies | | | | | | | | | | | |
| --- | --- | --- | --- | --- | --- | --- | --- | --- | --- | --- | --- |
| # | Study | Country | Design | Patients (n) | Inclusion criteria | RRT or ECMO | Antimicrobials | Quantification method | Modelling software | Covariate selection method | Covariates reported |
| 1 | Abdul-Aziz 2016 | Malaysia | Prospective PK study | 12 | Adult (≥ 18 years old) ICU patients prescribed doripenem for the treatment of sepsis | No | Doripenem | HPLC | NONMEM 7.3 | Biological plausibility | Yes |
| 2 | Al-Shaer 2020 | USA | Retrospective PK study | 230 | Paediatric dataset: ages 2 months to 18 years; adult dataset: 18 years or older, received cefepime, had cefepime concentrations reported as part of routine clinical care, admitted to the medical, surgical, cardiac, or neurological ICU | No | Cefepime | HPLC, LC-MS/MS | Pmetrics 1.5.2 | Not reported | Yes |
| 3 | Al-Shaer 2021 | USA | Prospective PK study | 10 | ≥ 18 years old, were admitted to the ICU, and received cefepime infusion while on CRRT | RRT | Cefepime | HPLC | Pmetrics 1.9.7 | Not reported | Yes |
| 4 | Al-Shaer 2022 | USA | Retrospective PK study | 125 | Dataset 1: adult ICU patients who received cefepime while on CVVH or CVVHD; dataset 2: adult patients admitted to the medical or surgical ICU, receiving CVVH or CVVHD, and received cefepime; dataset 3: ICU patients on RRT, received cefepime therapy, and had cefepime plasma concentration measured | RRT | Cefepime | HPLC, LC-MS/MS | Pmetrics 1.9.7 | Not reported | No |
| 5 | Alobaid 2016 | Australia | Prospective PK study | 19 | Age of ≥ 18 years, receiving meropenem, body mass index of ≥ 18.5 kg/m2 | No | Meropenem | HPLC-UV | Pmetrics | Biological plausibility | Yes |
| 6 | Alobaid 2017 | Australia | Prospective PK study | 37 | Age of ≥ 18 years, treatment with piperacillin, BMI ≥ 18.5 kg/m2 | No | Piperacillin | UHPLC-MS/MS | Pmetrics | Not reported | Yes |
| 7 | An 2023 | USA | Prospective PK study | 130 | Adult ICU patients prescribed intravenous meropenem as the standard of care | No | Meropenem | LC-MS/MS | NONMEM 7.4.3 | Biological plausibility | Yes |
| 8 | Asin-Prieto 2014 | Spain | Prospective PK study | 16 | Aged > 18 years and needing to be treated with CVVH for at least 20 hours | RRT | Piperacillin/tazobactam | HPLC–UV | NONMEM 7.2 | Not reported | Yes |
| 9 | Barreto 2023 | USA | Prospective PK study | 100 | Treated with cefepime at a stable dose and interval | No | Cefepime | IDMS | Monolix 2020R1 | Biological plausibility | Yes |
| 10 | Bastida 2020 | Spain | Prospective PK study | 54 | Presence of cirrhosis, age ≥ 18 years and clinical indication for treatment with meropenem | No | Meropenem | UHPLC | NONMEM 7.3 | Biological plausibility | Yes |
| 11 | Benitez-Cano 2020 | Spain | Prospective randomised PK trial | 31 | Age ≥ 18 years, diagnosis of nosocomial pneumonia and risk factors for MDR pathogens, GFR ≥  50 ml/min/1.73 m2 | No | Meropenem | HPLC | Pmetrics | Not reported | Yes |
| 12 | Bhalodi 2013 | USA | Prospective PK study | 25 | Received at least 3 doses of doripenem for any infection | No | Doripenem | HPLC | BigNPAG | Not reported | Yes |
| 13 | Boonpeng 2022 | Thailand | Prospective PK study | 52 | Age of ≥ 18 years, confirmed or suspected bacterial infection, intravenous meropenem therapy, hospitalization in a medical or surgical ICU | No | Meropenem | HPLC | NONMEM 7.4.3, Pirana 3.0.0 | Not reported | Yes |
| 14 | Braune 2018 | Germany | Prospective PK study | 19 | Aged > 18 years treated with meropenem and receiving SLED daytime | RRT | Meropenem | HPLC-UV | Pmetrics | Biological plausibility | Yes |
| 15 | Bue 2020 | Denmark | Prospective PK study | 10 | Patients treated with piperacillin/tazobactam and requiring RRT | RRT | Piperacillin/tazobactam | UHPLC | NONMEM 7.4.3, Pirana | Graphical evaluation, LRT | Yes |
| 16 | Buning 2021 | Netherlands | Retrospective PK study | 96 | Aged ≥18 years treated with IV ceftazidime for a proven or suspected clinically relevant P. Aeruginosa infection, and with at least one detectable ceftazidime serum concentration available during the course of therapy | No | Ceftazidime | LC-MS/MS | NONMEM 7.1.2 | Not reported | Yes |
| 17 | Burger 2018 | Switzerland | Prospective PK study | 86 | Prospectively enrolled in a therapeutic drug monitoring study, which aimed to assess the clinical utility of measuring antibiotic blood concentrations (n = 30) or were included in the institutional TDM programme (n = 56) | RRT | Meropenem | UPLC-MS, HPLC-UV | NONMEM 7.2, Xpose4 | Not reported | Yes |
| 18 | Burkhardt 2007 | Germany | Prospective PK study | 17 | Critically ill patients treated with ertapenem for early-onset VAP (≤4 days of mechanical ventilation). VAP diagnosis based on a new and persistent infiltrate on the chest radiograph, and two of the following three criteria: fever > 38.3°C, WBC count > 12 000 cells/mm3, and/or purulent tracheobronchial secretions | No | Ertapenem | HPLC | NONMEM 5 | Not reported | Yes |
| 19 | Carlier 2013 | Belgium | Prospective PK study | 13 | Admitted to the ICU and prescribed amoxicillin/clavulanic acid | No | Amoxicillin/clavulanic acid | UHPLC-MS/MS | NONMEM 6.1 | Not reported | Yes |
| 20 | Carlier 2014 | Belgium | Prospective PK study | 20 | Admitted to the ICU and prescribed cefuroxime | No | Cefuroxime | UHPLC-MS/MS | NONMEM 7.3 | Not reported | Yes |
| 21 | Chauzy 2019 | France | Prospective PK study | 11 | Hospitalized in the neurosurgery ICU with an EVD and with or without suspected meningitis/ventriculitis (> 5 leucocytes/mm3 and/or positive direct examination or positive culture) | No | Ceftaroline | LC-MS/MS | NONMEM 7.4 | Not reported | Yes |
| 22 | Chauzy 2022 | France | Prospective PK study | 18 | ICU patients aged ≥ 18 years, mechanically ventilated, presenting early-onset (i.e. During the 7 days following hospital admission) pneumonia caused by Gram-positive and/or Gram-negative bacteria and a creatinine clearance more than 80 ml/min/1.73 m2 estimated by the MDRD formula | No | Ceftaroline | LC–MS/MS | NONMEM 7.4 | Not reported | Yes |
| 23 | Cheatham 2014 | USA | Prospective PK study | 9 | Adult patients who were hospitalized in an intensive care unit, body mass index ≥ 40 kg/m2, antimicrobial therapy for a suspected or documented bacterial infection, central venous access, and an estimated creatinine clearance ≥ 50 ml/min | No | Meropenem | HPLC | Boomer 3.3.6 | Not reported | Yes |
| 24 | Chen 2020 | China | Retrospective PK study | 247 | Patients ≥18 years old; sex (male and female); patients who were administered imipenem after clinical diagnosis; patients without additional lifestyle risk factors, such as smoking; patients with or without ECMO | ECMO | Imipenem | UPLC-MS/MS | NONMEM 7.2 | Not reported | Yes |
| 25 | Cheng 2021a | International | Prospective PK study | 27 | ICU patients aged between 18 and 90 years old who were receiving piperacillin and tazobactam while undergoing ECMO for respiratory and/or cardiac dysfunction | ECMO, RRT | Piperacillin/tazobactam | UHPLC-MS/MS | Pmetrics | Biological plausibility | Yes |
| 26 | Cheng 2021b | International | Prospective PK study | 6 | Patients aged between 18–90 years, hospitalised in ICU and who were receiving cefepime whilst undergoing ECMO for respiratory and/or cardiac dysfunction | ECMO | Cefepime | HPLC-PDA | Pmetrics | Not reported | Yes |
| 27 | Cheng 2022 | International | Prospective PK study | 14 | ICU patients aged 18–90 years who were receiving ceftriaxone whilst undergoing ECMO for respiratory and/or cardiac dysfunction | ECMO, RRT | Ceftriaxone | UHPLC-MS/MS | Pmetrics | Biological plausibility | Yes |
| 28 | Chung 2017 | USA | Prospective PK study | 20 | Adult patients 18 to 65 years of age who were obese (BMI ≥ 40 kg/m2 or TBW ≥ 45.5 kg over their ideal body weight [IBW]) and hospitalized in an ICU or non-ICU | No | Doripenem | UPLC-MS/MS | NONMEM 7.4 | Not reported | Yes |
| 29 | Cojutti 2021 | Italy | Retrospective PK study | 74 | Critically ill patients who were admitted to ICU and received continuous infusion meropenem for empirical or targeted treatment of Gram-negative-related infections | No | Meropenem | LC–MS/MS | Pmetrics | Biological plausibility | Yes |
| 30 | Conil 2007 | France | Prospective PK study | 50 | ICU burns patients in the secondary phase of their burn injuries with local infections or sepsis (empirically or targeted) prescribed ceftazidime | No | Ceftazidime | HPLC | NONMEM, Visual-NM | Not reported | Yes |
| 31 | Couffignal 2014 | France | Prospective PK study | 63 | Male or female over 18 years of age; use of mechanical ventilation for >48 h; clinical suspicion of VAP (new or persistent radiological infiltrate and one of the following criteria: purulent tracheal aspiration or temperature ≥ 38.3°C or leucocytosis >10 000 ml−1); VAP with high risk of multiresistant bacteria (at least 6 days of mechanical ventilation or antibiotic treatment within 15 days) | No | Imipenem | HPLC | Monolix 4.1.2 | Previously published covariates | Yes |
| 32 | Crandon 2011 | USA | Prospective PK study | 26 | At least 18 years of age and had received at least 3 consecutive doses of meropenem | No | Meropenem | HPLC | Not reported | Not reported | Yes |
| 33 | De Jongh 2008 | Belgium | Prospective randomised PK trial | 17 | (i) a high probability of infection from nosocomial origin [body temperature >38 or <35.5°C not induced by external factors; leucocytosis or leucopenia; one or several suspected infection foci (based on X-ray pathognomonic image, purulent sputum, white blood cells in urine, or other accepted clinical sign)]; and (ii) no suspicion of an infection by Pseudomonas spp. Or another temocillin-resistant bacteria | No | Temocillin | HPLC | WinNonMix | Not reported | No |
| 34 | de Velde 2020 | Switzerland | Retrospective PK study | 26 | Suspected or documented severe bacterial infection and age 18–60 years | No | Imipenem/cilastatin | HPLC-UV | NONMEM 7.2, Pmetrics | Not reported | Yes |
| 35 | Delattre 2012 | Belgium | Prospective PK study | 88 | ICU patients diagnosed with severe sepsis or septicshock at their ICU admission or during their ICU stay. Severe sepsis and septic shock were defined according to standard criteria | No | Piperacillin, ceftazidime, cefepime, meropenem, amikacin | HPLC | NONMEM 4 | Biological plausibility | Yes |
| 36 | Dhaese 2018 | Belgium | Prospective PK study | 110 | Patients who were admitted to the surgical ICU and received continuous infusion of piperacillin | No | Piperacillin/tazobactam | UHPLC-MS/MS | Pmetrics 1.5.0 | Not reported | Yes |
| 37 | Dhaese 2019a | Belgium | Prospective PK study | 17 | Admitted to the surgical or medical ICU and received piperacillin/tazobactam in continuous infusion | No | Piperacillin/tazobactam | UPLC-MS/MS | Pmetrics 1.5.2 | Biological plausibility | Yes |
| 38 | Dhaese 2019b | Belgium | Prospective PK study | 68 | Admitted to the surgical ICU and received meropenem as a continuous infusion | No | Meropenem | UPLC–MS/MS | Pmetrics 1.5.0 | Not reported | Yes |
| 39 | Dinh 2022 | Vietnam | Prospective PK study | 24 | Admitted to the intensive care unit and got imipenem per clinical indication | No | Imipenem | HPLC-UV | Monolix 2019R1 | Previously published covariates | Yes |
| 40 | Dreesen 2022 | Belgium | Prospective PK study | 33 | All adult patients admitted to the ICU with pneumonia and treated with ceftriaxone | No | Ceftriaxone | HPLC-MS/MS | NONMEM 7.4 | Biological plausibility | Yes |
| 41 | Economou 2019 | Australia | Prospective PK study | 6 | (i) age ≥18 years; (ii) admission to an ICU; (iii) clinical indication for TCC (only the combination formulation was available in Australia at the time of study); and (iv) receiving EDDf | RRT | Ticarcillin | UHPLC-MS/MS | Pmetrics 1.5.1 | Biological plausibility | Yes |
| 42 | Ehmann 2019 | Germany | Prospective PK study | 48 | Presence of severe infection (confirmed or suspected by clinical assessment), age ≥ 18 years and therapy with meropenem | ECMO, RRT | Meropenem | UHPLC-MS/MS | NONMEM 7.3 | Biological plausibility, previously published | Yes |
| 43 | Eisert 2021 | Germany | Prospective PK study | 32 | Septic shock according to the Sepsis-1 definition, being aged ≥ 18 years and necessity for treatment with meropenem, as determined by the treating physicians | RRT | Meropenem | HPLC-UV | NONMEM 7.3 | Not reported | Yes |
| 44 | Eyler 2014 | USA | Prospective PK study | 8 | ≥18 years of age, receiving CVVHDF, and prescribed ertapenem | RRT | Ertapenem | HPLC-MS/MS | NONMEM 7 | Not reported | Yes |
| 45 | Felton 2014a | UK | Prospective PK study | 18 | Intubated patients who received piperacillin–tazobactam for suspected or documented pulmonary infection | RRT | Piperacillin/tazobactam | LC-MS/MS | Pmetrics 1.1.3 | Not reported | No |
| 46 | Felton 2014b | UK | Retrospective PK study | 146 | Study 1: 76 patients undergoing abdominal or thoracic surgery who received 2 g of piperacillin over 30 min; Study 2: 12 hospitalized patients, who had received 3 g of piperacillin over 4 h every 8 h were included; Study 3: 40 ventilated patients with ventilator-associated pneumonia, administered piperacillin (12 g or 16 g) by continuous infusion | No | Piperacillin/tazobactam | Not reported | Pmetrics 1.1.1 | Not reported | Yes |
| 47 | Felton 2018 | UK | Retrospective PK study | 53 | Study 1: 13 critically ill, mechanically ventilated patients (4 patients were excluded from the analysis as they were receiving renal replacement therapy); Study 2: 40 critically ill patients with VAP | No | Piperacillin/tazobactam | HPLC, LC–MS/MS | Pmetrics 1.4 | Not reported | Yes |
| 48 | Fillatre 2021 | France | Prospective PK study | 42 | (i) ECMO patients requiring antimicrobial therapy for sepsis;23 (ii) use of piperacillin/tazobactam; and (iii) the presence of an arterial line allowing iterative blood sampling for the PK study | ECMO, RRT | Piperacillin/tazobactam | HPLC-UV | Monolix 4.3.2 | Not reported | Yes |
| 49 | Fournier 2018 | Switzerland | Prospective PK study | 21 | All burn patients admitted to the Burn Centre who received a course of intravenous amoxicillin administered either alone or in combination with clavulanic acid | No | Amoxicillin/clavulanic acid | UPLC-MS/MS | NONMEM 7.1.0 | Not reported | Yes |
| 50 | Fratoni 2022 | USA | Prospective PK study | 9 | Patients with presumed ARC in the medical, surgical, and neurosurgical icus (as defined by Cockcroft-Gault calculated creatinine clearance ≥130 ml/min within 24 h of dosing) were considered eligible. Additional inclusion criteria were: (i) male or female aged 18–54 years old, (ii) APACHE II score ≥10 and ≤32, and (iii) a documented or presumed infection as defined by receipt of another antibiotic. | No | Imipenem/cilastatin/relebactam | LC-MS/MS | Pmetrics | Not reported | Yes |
| 51 | Frippiat 2015 | Belgium | Prospective PK study | 55 | Age >18 years; diagnosis of late-onset (>5 days after admission) ventilator-associated pneumonia or hospital-acquired pneumonia requiring mechanical ventilation; and glomerular filtration rate (GFR) ≥30 ml/min (calculated according to the four-variable Modification of Diet in Renal Disease formula or by measurement of creatinine clearance based on 24 h urine) or acute kidney injury with indication for CVVH | RRT | Meropenem | UPLC-UV | NONMEM 7.2 | Not reported | Yes |
| 52 | Garot 2011 | France | Prospective PK study | 54 | At least 18 years of age, hospitalized in the ICU for sepsis, severe sepsis or septic shock, and treated with ceftriaxone | RRT | Ceftriaxone | HPLC | NONMEM 6 | Biological plausibility | Yes |
| 53 | Georges 2009 | France | Prospective randomised PK trial | 72 | (i) an inpatient stay in the ICU, (ii) over 18 years old, and (iii) presenting with P. Aeruginosa nosocomial pneumonia or bacteremia with a strain thought to be sensitive to ceftazidime | No | Ceftazidime | HPLC-UV | NONMEM | Biological plausibility | Yes |
| 54 | Gijsen 2022 | Belgium | Prospective PK study | 58 | All adult patients admitted to the ICU, treated with meropenem and having severe sepsis or septic shock (as defined according to the definitions applicable at the time of the study) at the start of meropenem therapy were screened for inclusion | No | Meropenem | UHPLC-MS/MS | NONMEM 7.4 | Previously published, expert consensus | Yes |
| 55 | Goncalves-Pereira 2014 | Portugal | Prospective PK study | 15 | Infected critically ill patients requiring intravenous meropenem (by decision of the attending physician), admitted to the intensive care unit (ICU) between May of 2009 and May of 2010, were recruited, irrespectively of comorbidities or of renal function | No | Meropenem | HPLC-UV | WinNonlin 5.0.1 | Biological plausibility | Yes |
| 56 | Grensemann 2020 | Germany | Prospective PK study | 19 | Receiving meropenem for clinical indication and required CVVHD | RRT | Meropenem | HPLC-DAD | NONMEM 7.4 | Not reported | No |
| 57 | Hahn 2021 | South Korea | Prospective PK study | 26 | Critically ill patients aged 19 years or older who were admitted to the CCU for venoarterial ECMO and were prescribed piperacillin/tazobactam | ECMO, RRT | Piperacillin/tazobactam | LC-MS/MS | NONMEM 7.4 | Biological plausibility | Yes |
| 58 | Hanberg 2018 | Denmark | Prospective PK study | 10 | Patients on ECMO treatment who were treated with meropenem | ECMO, RRT | Meropenem | UHPLC-UV | NONMEM 7.3 | Not reported | Yes |
| 59 | Heffernan 2022 | Australia | Retrospective PK study | 36 | Study 1: Twelve adult patients in the ICU with severe sepsis and who received ceftriaxone; Study 2: 17 patients, not clear what inclusion criteria; Study 3: adult patients (age ≥18 years) admitted to the ICU receiving ceftriaxone intravenously for the treatment of sepsis with an EVD were eligible | No | Ceftriaxone | UHPLC-MS/MS | Pmetrics 1.5.2 | Biological plausibility | Yes |
| 60 | Idoate Grijalba 2019 | France | Retrospective PK study | 80 | Aged over 18 years, admitted to ICU, treated with empirical or targeted therapy with meropenem, and had received at least 3 doses of meropenem prior to monitoring | No | Meropenem | HPLC | Pmetrics | Not reported | Yes |
| 61 | Isla 2008 | Spain | Prospective PK study | 20 | (i) age >18 years; (ii) treatment with RRT for >1 day for ≥20 h/day; and (iii) isolated or expected causative pathogen susceptible to meropenem | RRT | Meropenem | HPLC | NONMEM 5 | Not reported | Yes |
| 62 | Jacobs 2018 | Belgium | Retrospective PK study | 215 | (i) diagnosis of sepsis or septic shock according to standard criteria (35), (ii) therapy with a standard dosage regimen of a broad-spectrum β-lactam and (iii) measured CLCr of ≥ 120 ml/min (so that all patients close to the threshold of ARC could be included in the study) on the day of TDM | No | Meropenem, cefepime, ceftazidime, piperacillin | HPLC-UV | Pmetrics | Biological plausibility | Yes |
| 63 | Jager 2020 | Australia | Retrospective PK study | 35 | Study 1: age between 18 and 80 years old, known or suspected infection produced by MSSA, hypoalbuminaemia arbitrarily defined as albumin plasma levels ≤ 32 g/L and without severe renal dysfunction arbitrarily defined as plasma creatinine concentration < 170 µmol/L; Study 2: ≥18 years old, receiving one of the selected study antibiotic(s), would have received at least four doses of antibiotic by the time of sampling and were expected to remain on the treatment for the next 24 h | No | Flucloxacillin | HPLC-UV | NONMEM 7.1.2 | Biological plausibility | Yes |
| 64 | Jaruratanasirikul 2015 | Thailand | Prospective PK study | 9 | (i) > 18 years of age and (ii) with a diagnosis of severe sepsis or septic shock, either at admission or during the ICU stay | No | Meropenem | HPLC | NONMEM 7.2 | Not reported | Yes |
| 65 | Jaruratanasirikul 2019 | Thailand | Prospective PK study | 10 | Aged ≥ 18 years with cardiopulmonary failure and were supported by ECMO receiving a 1-h infusion of 0.5 g imipenem/cilastatin diluted in 100 ml of normal saline solution, delivered via infusion pump at a constant flow rate, every 6 h for the treatment of severe infections for 14 days | ECMO | Imipenem | HPLC | WinNonlin 1.1 | Not reported | Yes |
| 66 | Jeon 2014 | South Korea | Prospective PK study | 50 | Burns ranging from 1% to 81% of their TBSA who were treated with piperacillin-tazobactam | No | Piperacillin/tazobactam | LC-MS/MS | NONMEM 7.2 | Not reported | Yes |
| 67 | Jonckheere 2016 | Belgium | Prospective PK study | 20 | Patients during the first 3 days of cefepime therapy | RRT | Cefepime | LC-MS/MS | NONMEM 7.3 | Not reported | Yes |
| 68 | Kang 2022 | South Korea | Prospective PK study | 13 | Adult patients (≥18 years) receiving VA ECMO and concomitantly receiving meropenem | ECMO, RRT | Meropenem | LC-MS | NONMEM 7.4.1, Pirana 2.9.7 | Biological plausibility | Yes |
| 69 | Kanji 2018 | Canada | Prospective PK study | 34 | 18 years of age or older, admitted to 1 of the 2 participating icus, and receiving SLED and any dose of piperacillin/tazobactam | RRT | Piperacillin/tazobactam | LC-MS/MS | Pmetrics | Not reported | Yes |
| 70 | Kees 2016 | Germany | Prospective PK study | 32 | Adult patients (≥18 years) who received continuous infusion of meropenem and were not on renal replacement therapy were eligible for study participation | No | Meropenem | HPLC | NONMEM 7.3 | Biological plausibility | Yes |
| 71 | Kim 2022 | South Korea | Prospective PK study | 38 | Nosocomial infections, empirical management of septic shock from an unknown source, and prophylactic administration for patients undergoing ECMO | ECMO, RRT | Piperacillin/tazobactam | LC-MS/MS | NONMEM 7.5 | Biological plausibility | Yes |
| 72 | Klastrup 2020 | Denmark | Prospective PK study | 78 | Critically ill patients treated empirically or targeted with piperacillin-tazobactam, administered as continuous infusion | No | Piperacillin/tazobactam | UHPLC | NONMEM 7.4.3 | Not reported | Yes |
| 73 | Kois 2022 | USA | Prospective PK study | 6 | ≥18 years old, receiving VA or VV ECMO, and receiving cefepime as part of their standard-of-care antibiotic regimen | ECMO, RRT | Cefepime | LC-MS/MS | Pmetrics | Not reported | No |
| 74 | Konig 2017 | Germany | Prospective PK study | 16 | The inclusion criteria were age ≥18 years and antibiotic treatment with ceftazidime in septic patients receiving SLED | RRT | Ceftazidime | HPLC-UV | Pmetrics | Biologic plausibility | Yes |
| 75 | Kothekar 2020 | India | Prospective PK study | 25 | We included patients aged 18–70 years of either sex, with known or suspected severe sepsis or septic shock admitted to the ICU and receiving meropenem 1000 mg 3-h extended infusion eight hourly as a standard of care. Severe sepsis and septic shock were diagnosed according to the American–European Consensus Conference criteria | No | Meropenem | HPLC | WinNonlin | Not reported | No |
| 76 | Krueger 1998 | Germany | Prospective PK study | 9 | Not reported | RRT | Meropenem | HPLC-UV | MODFIT | Not reported | No |
| 77 | Kumta 2022 | International | Prospective PK study | 8 | (i) age 18 to 85 years; (ii) EVD in situ; (iii) receiving meropenem treatment; and (iv) a diagnosis of ventriculitis or extracranial infection | No | Meropenem | UHPLC-MS/MS | Pmetrics 1.5.2 | Not reported | Yes |
| 78 | Layios 2022 | Belgium | Prospective PK study | 32 | Age > 18; diagnosis of VAP or HAP requiring mechanical ventilation with a documented pathogen showing temocillin in vitro sensitivity of ≤8 mg/L; and creatinine clearance based on 24-h urine output collection and measurement ≥30 ml/min/1.73 m2 | No | Temocillin | UHPLC-MS/MS | NONMEM 7.4.0 | Not reported | Yes |
| 79 | Lee 2021a | South Korea | Prospective PK study | 30 | Patients (aged ≥19) who underwent ECMO for respiratory and/or cardiac dysfunction and who received meropenem for treatment or prophylaxis | ECMO, RRT | Meropenem | HPLC-MS/MS | NONMEM 7.5 | Clinical relevance | Yes |
| 80 | Lee 2021b | South Korea | Prospective PK study | 26 | Empirical management of sepsis from unknown source, nosocomial infections, and prophylactic administration for patients undergoing ECMO | ECMO, RRT | Meropenem | HPLC-MS/MS | NONMEM 7.5 | Clinical relevance | Yes |
| 81 | Leegwater 2020 | Netherlands | Prospective PK study | 55 | Patients aged ≥18 years were eligible for inclusion if ceftriaxone therapy was initiated in the ICU | No | Ceftriaxone | LC-MS/MS | NONMEM 7.4.1, Pirana 2.9.8 | Biological plausibility, previously published | Yes |
| 82 | Li 2019 | Belgium | Retrospective PK study | 20 | Study 1: 1) adult; 2) admission to ICU; 3) treatment with imipenem and RRT simultaneously; Study 2: All patients 18 years or older in the Firefighters’ Burn Center ICU at Regional One Health receiving antimicrobials for any infectious indication and requiring high-dose CVVH were eligible for inclusion | RRT | Imipenem | HILIC-MS, HPLC-UV | NONMEM 7.3, PSN 4.60, Pirana 2.9.6 | Not reported | Yes |
| 83 | Li 2020 | China | Prospective PK study | 30 | Adult, admission to the intensive care unit, and simultaneous treatment with imipenem and RRT | RRT | Imipenem | HPLC-UV | Phoenix 8.0 | Not reported | Yes |
| 84 | Liebchen 2021 | Germany | Retrospective PK study | 155 | Patients with characteristics within the 90% range of the characteristics of the original dataset used for the PK model development were selected for the evaluation. | No | Meropenem | LC-MS/MS | Not reported | Not reported | Yes |
| 85 | Lipman 1999 | Australia | Prospective PK study | 10 | Critically ill patients ranging in age from 18 to 75 years for whom the staff intensive care specialist deemed cefepime to be appropriate therapy were enrollable if they had an infected site as defined by clinical suspicion with or without positive culture results, systemic inflammatory response syndrome, and serum creatinine level of <0.1 mmol/L | No | Cefepime | HPLC | WinNonlin | Not reported | No |
| 86 | Lipman 2001 | Australia | Prospective PK study | 12 | (A) 18-75 years of age; (b) infected site as defined by clinical suspicion with or without positive culture results; (c) systemic inflammatory response syndrome; (d) informed consent given by patient or next of kin; (e) normal renal function; (f) an intra-arterial cannula in situ | No | Cefpirome | HPLC | WinNonlin | Not reported | No |
| 87 | Mathew 2016 | India | Prospective PK study | 35 | Adult patients admitted to the ICU and initiated on meropenem at a dose of 1000 mg (twice or thrice daily) were recruited into the study after obtaining a written informed consent from the patient or the closest relative of the patient. Meropenem was initiated for the treatment of sepsis, pneumonia, polytrauma, complicated urinary tract infection, or as empirical therapy | No | Meropenem | HPLC | Pmetrics 1.4.1 | Not reported | Yes |
| 88 | Mattioli 2016 | Italy | Prospective PK study | 27 | Patients admitted to icus who developed a Klebsiella pneumoniae (KP) nosocomial infection treated with meropenem alone or in combination depending on the resistance profile of the bacterial strain, meropenem administration for at least 2 days, and bacteremia confirmed by at least one positive blood culture | No | Meropenem | HPLC | NONMEM 7.2 | Not reported | Yes |
| 89 | Minichmayr 2018 | Germany | Retrospective PK study | 195 | Adult critically ill patients, who received continuous infusion of meropenem | No | Meropenem | HPLC-UV | NONMEM 7.3 | Not reported | Yes |
| 90 | Mueller 2002 | Germany | Prospective PK study | 8 | Age of >18 years, acute renal failure treated by CVVHD, anuria (<100 ml of urine/day), and treatment with piperacillin-tazobactam | RRT | Piperacillin/tazobactam | HPLC-UV | Topfit 2.0 | Not reported | No |
| 91 | Murinova 2022 | Czech Republic | Retrospective PK study | 144 | Age ≥18 years, not receiving dialysis, receiving intravenous meropenem to treat a serious bacterial infection defined as the development of potentially life-threatening clinical manifestations, and having at least one measured meropenem serum level in the course of meropenem therapy | No | Meropenem | LC-MS/MS | Monolix 2021R1 | Not reported | Yes |
| 92 | Nandy 2010 | USA | Retrospective PK study | 285 | In the phase 2 study of patients with complicated UTI or pyelonephritis, doripenem at 250 to 500 mg was infused over 60 min every 8 h. In the two phase 3 studies of patients with nosocomial pneumonia, doripenem at 500 mg was infused over either 1 or 4 h every 8 h. | No | Doripenem | LC-MS/MS | NONMEM V | Not reported | Yes |
| 93 | Nicasio 2009 | USA | Prospective PK study | 26 | Adult patients (≥18 years old) in the ICU, who were placed on the VAP clinical pathway and prescribed cefepime | No | Cefepime, vancomycin, tobramycin | HPLC | MM-USC*PACK | Not reported | Yes |
| 94 | Niibe 2020 | Japan | Prospective PK study | 21 | Admitted to the ICU between July 2016 to March 2018, inclusive, and who were treated with an intravenous infusion of meropenem and were receiving CVVHDF | RRT | Meropenem | HPLC | Phoenix 7.0 | Not reported | Yes |
| 95 | Niibe 2022 | Japan | Prospective PK study | 12 | Age ≥18 years, both medical and surgical origin of ICU admission between July 2016 to September 2017, and treatment with an intravenous infusion of meropenem initiated after ICU admission | No | Meropenem | HPLC | Phoenix 7.0 | Not reported | Yes |
| 96 | Nonoshita 2020 | Japan | Prospective PK study | 21 | In ICU treated with doripenem who gave written informed consent obtained from either the patients or their legally authorized representatives | RRT | Doripenem | HPLC | NONMEM 7.3.0 | Clinical plausibility | Yes |
| 97 | O'Jeanson 2021 | France | Retrospective PK study | 58 | Known or suspected infection of a critically ill patient in the medical ICU and treated with meropenem | RRT | Meropenem | UHPLC-UV | NONMEM 7.4.1 | Biological plausibility | Yes |
| 98 | Öbrink-Hansen 2015 | Denmark | Prospective PK study | 15 | Critically ill patients with known or suspected septic shock who required noradrenaline infusion and who were prescribed piperacillin-tazobactam by the treating physician were eligible for the study. All patients had an arterial catheter. | No | Piperacillin/tazobactam | UHPLC | NONMEM 7.2 | Not reported | Yes |
| 99 | Ollivier 2019 | France | Prospective PK study | 21 | (i) the patient was ≥18 years of age, (ii) the patient had a first episode of sepsis (defined as presumed or confirmed infection with new organ dysfunction in the previous 48 h), (iii) the patient had been treated with ceftriaxone for less than 24 h at the time of the assessment, and (iv) the patient had an expected ICU stay of greater than 72 h | No | Ceftriaxone | HPLC-UV | Monolix | Not reported | Yes |
| 100 | Onichimowski 2020 | Poland | Prospective PK study | 19 | Age 18–80 years; medical/surgical ICU; treatment with licensed doses of meropenem; clinical indications for RRT due to AKI | RRT | Meropenem | HPLC | NONMEM 7.4.1 | Biological plausibility | Yes |
| 101 | Padulles Zamora 2019 | Spain | Prospective PK study | 12 | (i) sepsis with acute renal failure requiring RRT with high-adsorbent membranes for >48 h; (ii) age ≥18 years; and (iii) isolated or expected causative pathogen susceptible to meropenem, prescribed at the discretion of the treating physician | RRT | Meropenem | UHPLC-MS/MS | NONMEM 7.3 | Not reported | Yes |
| 102 | Pokem 2022 | Belgium | Prospective PK study | 19 | Patients with septic shock, >18 years old, diagnosed with an intra-abdominal infection caused by a pathogen expected to be susceptible to temocillin | No | Temocillin | HPLC-MS/MS | Pmetrics 1.5.1 | Biological plausibility | Yes |
| 103 | Por 2021 | USA | Prospective PK study | 23 | Not reported | RRT | Imipenem | HPLC-UV | Pumas 1.05 | Previously published | Yes |
| 104 | Rahbar 2016 | USA | Prospective PK study | 27 | Age ≥18 years, admission to the Emory surgical intensive care unit service for greater than 24 hours, and a diagnosis of sepsis that necessitated empiric antimicrobial therapy | No | Doripenem | HPLC | BigNPAG | Not reported | No |
| 105 | Robatel 2003 | Switzerland | Prospective PK study | 15 | Treated by CVVHDF and receiving meropenem since at least 1 day | RRT | Meropenem | HPLC-UV | Kinetica | Not reported | No |
| 106 | Roberts 2009a | Australia | Prospective randomised PK trial | 13 | Critically ill adults; known or suspected sepsis; treating physician deemed piperacillin–tazobactam appropriate therapy | No | Piperacillin/tazobactam | HPLC-UV | Scientist 2.0 | Not reported | No |
| 107 | Roberts 2009b | Australia | Prospective PK study | 10 | Known or suspected sepsis11 of a critically ill patient and normal renal function (defined as plasma creatinine concentration <120 µmol/L). Clinical indications for meropenem included nosocomial pneumonia, soft tissue infection, intra-abdominal sepsis and empirical therapy for sepsis without proven source | No | Meropenem | HPLC-UV | NONMEM 6.1 | Not reported | Yes |
| 108 | Roberts 2010 | Australia | Prospective randomised PK trial | 16 | Patients admitted to the Critical Care Unit with known or suspected sepsis as defined previously and with normal renal function | No | Piperacillin/tazobactam | HPLC-UV | NONMEM 6.1 | Not reported | Yes |
| 109 | Roberts 2013 | International | Retrospective PK study | 31 | Critically ill adult patients with nosocomial pneumonia and a clinical indication for doripenem were included | No | Doripenem | LC-MS/MS | NONMEM 6.1 | Not reported | Yes |
| 110 | Roberts 2014 | Australia | Prospective PK study | 12 | Critically ill adult patients with AKI receiving CVVHDF and a clinical indication for doripenem | RRT | Doripenem | HPLC-MS/MS | S-ADAPT 1.57 | Not reported | Yes |
| 111 | Roberts 2015a | Australia | Prospective PK study | 24 | AKI and at least one of the following: oliguria, hyperkalemia, severe acidemia, an elevated plasma urea or creatinine concentration, or clinically significant organ edema | RRT | Meropenem, piperacillin/tazobactam, ciprofloxacin, vancomycin | LC-MS/MS | NONMEM 6.1 | Not reported | Yes |
| 112 | Roberts 2015b | Australia | Prospective PK study | 30 | (I) age 18–80 years; and (ii) receiving cefazolin as prophylaxis for skin and soft tissue and bone infections after major trauma | No | Cefazolin | HPLC | Pmetrics | Biological plausibility | Yes |
| 113 | Roelofsen 2023 | Netherlands | Prospective PK study | 92 | ≥18 years and admitted to the ICU with an expected stay of >72 hours | No | Cefotaxime | LC-MS/MS | NONMEM 7.4.2 | Not reported | Yes |
| 114 | Rohani 2022 | USA | Retrospective PK study | 70 | Critically ill patients with HAP admitted to the medical ICU were enrolled between 30 June 2018 and 1 March 2021 | No | Meropenem | LC-MS/MS | Pmetrics 1.9.7 | Not reported | Yes |
| 115 | Roos 2006 | Australia | Retrospective PK study | 13 | Patients were enrolled if they had a serum creatinine concentration of <0.1 mmol/L | No | Cefepime | HPLC | NONMEM 5 | Not reported | Yes |
| 116 | Roos 2007 | Australia | Prospective PK study | 12 | ICU patients; cefpirome; serum creatinine concentration less than 0.1 mmol/l | No | Cefpirome | HPLC | NONMEM 5 | Not reported | Yes |
| 117 | Sakka 2007 | Germany | Prospective randomised PK trial | 20 | ICU-acquired pneumonia (duration of mechanical ventilation of >3 days); normal renal function | No | Imipenem | LC-MS/MS | Pmetrics, WinNonlin | Not reported | Yes |
| 118 | Sanches 2022 | Brazil | Retrospective PK study | 24 | Aged >18 years, with a confirmed or suspected infection with indications for use of the antibiotic piperacillin/tazobactam | No | Piperacillin/tazobactam | HPLC-UV | Pmetrics 1.5.0 | Biological plausibility | Yes |
| 119 | Selig 2022a | USA | Retrospective PK study | 19 | Not reported | No | Piperacillin/tazobactam | HPLC-UV | Pumas 1.1 | Not reported | Yes |
| 120 | Selig 2022b | USA | Retrospective PK study | 23 | Not reported | RRT | Meropenem | HPLC-UV | Pumas 1.05 | Not reported | Yes |
| 121 | Shekar 2014 | Australia | Prospective PK study | 11 | Eligible patients ≥18 years of age and receiving meropenem during their ECMO therapy were recruited. | ECMO, RRT | Meropenem | HPLC | NONMEM 7.3 | Biological plausibility | Yes |
| 122 | Shotwell 2016 | USA | Prospective PK study | 68 | Patients ages 18 years old or older who were receiving piperacillin-tazobactam with concomitant RRT in the ICU were included. | RRT | Piperacillin/tazobactam | HPLC | NLME | Not reported | Yes |
| 123 | Sime 2019 | Australia | Prospective PK study | 12 | ICU patients, aged ≥18 years, were enrolled if diagnosed with a systemic infection known or suspected to be caused by a bacterium susceptible to ceftolozane-tazobactam | No | Ceftolozane/tazobactam | UHPLC-MS/MS | Pmetrics 1.5.2 | Biological plausibility, regression analysis | Yes |
| 124 | Sime 2020 | Australia | Prospective PK study | 6 | Adult patients (≥18 years) admitted to ICU who were prescribed RRT were enrolled if diagnosed with systemic infection known or suspected to be caused by an organism susceptible to ceftolozane-tazobactam. | RRT | Ceftolozane/tazobactam | UHPLC-MS/MS | Pmetrics | Biological plausibility | Yes |
| 125 | Sime 2021 | Australia | Prospective PK study | 10 | Adult patients (≥18 years) admitted to the ICU were enrolled if they had an indwelling external ventricular drain (EVD) and informed consent was obtained | No | Ceftolozane/tazobactam | UHPLC-MS/MS | Pmetrics 1.5.2 | Biological plausibility | Yes |
| 126 | Stein 2019 | USA | Prospective PK study | 10 | Critically ill (APACHE II > 10) adult patients being treated with ceftazidime/avibactam | No | Ceftazidime/avibactam | LC-MS/MS | Pmetrics | Not reported | Yes |
| 127 | Sturm 2014 | USA | Prospective PK study | 9 | Age ≥ 18 years; admitted to trauma-surgical ICU; BMI 40 kg/m2 or higher; piperacillin-tazobactam started for empiric or directed therapy; available intravenous access | No | Piperacillin/tazobactam | HPLC | Not reported | Not reported | No |
| 128 | Sukarnjanaset 2019 | Thailand | Prospective PK study | 48 | The inclusion criteria were (i) age ≥ 15 years, (ii) treatment with piperacillin/tazobactam, and (iii) sepsis defined according to the third international consensus definitions for sepsis and septic shock (Sepsis-3) | No | Piperacillin/tazobactam | HPLC | NONMEM 7.4.1 | Not reported | Yes |
| 129 | Swartling 2022 | Sweden | Prospective PK study | 51 | Patients above the age of 18 years admitted to the icus and treated with cefotaxime for a proven or suspected infection were included in the study. Patients were included within 24 h from initiation of cefotaxime treatment | No | Cefotaxime | LC-MS/MS | NONMEM 7.4 | Clinically available covariates | Yes |
| 130 | Tamme 2015 | Estonia | Prospective PK study | 9 | Severe sepsis or septic shock with AKI deemed by the treating clinician to require RRT, arterial line in situ | RRT | Doripenem | UHPLC-MS/MS | NONMEM 7.3.0 | Biological plausibility | Yes |
| 131 | Tegeder 1997 | Germany | Prospective PK study | 12 | Thirteen consecutive patients who were admitted to the surgical intensive care unit, who were treated with CVVH for AKI, and who were receiving imipenem-cilastatin for the treatment of a severe life-threatening infection were enrolled in this study | RRT | Imipenem | HPLC | Topfit | Not reported | No |
| 132 | Tegeder 1999 | Germany | Prospective PK study | 9 | Patients of the surgical ICU with severe life-threatening infections and acute renal failure were enrolled in this study provided they were treated with meropenem and undergoing CVVH | RRT | Meropenem | HPLC | Micromath Scientist | Not reported | No |
| 133 | Thuy 2018 | Vietnam | Prospective PK study | 30 | Patients admitted to ICU or general medicine department | No | Meropenem | HPLC | Monolix 2016 | Not reported | Yes |
| 134 | Truong 2022 | Vietnam | Prospective PK study | 27 | Aged ≥18 years with meropenem administration, ICU stay of >24 h, and a urine catheter in situ | No | Meropenem | HPLC-UV | Pmetrics | Clinical plausibility | Yes |
| 135 | Tsai 2016a | Australia | Prospective PK study | 9 | (i) Australian Indigenous; (ii) ≥18 years of age; (iii) confirmed or suspected severe sepsis within the previous 48 h; (iv) prescribed meropenem; and (v) an arterial line in situ | No | Piperacillin/tazobactam | UHPLC-MS/MS | Pmetrics | Not reported | Yes |
| 136 | Tsai 2016b | Australia | Prospective PK study | 11 | (i) Australian Indigenous; (ii) ≥18 years of age; (iii) confirmed or suspected severe sepsis within the previous 48 h; (iv) prescribed meropenem; and (v) an arterial line in situ | No | Meropenem | HPLC-UV | Pmetrics | Not reported | Yes |
| 137 | Udy 2015 | Australia | Prospective PK study | 48 | Between 18 and 80 years of age and were receiving piperacillin-tazobactam for the treatment of sepsis (defined as presumed or confirmed nosocomial infection while manifesting a systemic inflammatory response syndrome) | No | Piperacillin | HPLC-UV | NONMEM 7.1 | Not reported | Yes |
| 138 | Ulldemolins 2010 | Australia | Prospective PK study | 10 | Age between 18 and 80 years old, known or suspected infection produced by MSSA, hypoalbuminaemia arbitrarily defined as albumin plasma levels ≤32 g/L | No | Flucloxacillin | HPLC-UV | NONMEM 6.1 | Not reported | Yes |
| 139 | Ulldemolins 2015 | Spain | Prospective PK study | 30 | Age of ≥18 years, a diagnosis of septic shock by the criteria of the Surviving Sepsis Campaign guidelines, RRT, and an indication for treatment with meropenem | RRT | Meropenem | LC-MS/MS | NONMEM 7.3 | Biological plausibility | Yes |
| 140 | Ulldemolins 2016 | Spain | Prospective PK study | 19 | Age ≥18 years, MODS including septic shock diagnosed by the Surviving Sepsis Campaign guidelines criteria AKI requiring CVVHDF and clinical indication for piperacillin | RRT | Piperacillin/tazobactam | LC-MS/MS | NONMEM 7.3 | Biological plausibility | Yes |
| 141 | Ulldemolins 2021 | Spain | Prospective PK study | 8 | Inclusion criteria were age ≥18 years, septic shock diagnosed by the Surviving Sepsis Campaign guidelines criteria, AKI requiring CVVHDF, and clinical indication for ceftriaxone. | RRT | Ceftriaxone | LC-MS/MS | NONMEM 7.3 | Biological plausibility | Yes |
| 142 | Van Dalen 1986 | Netherlands | Prospective PK study | 20 | All patients were on mechanical ventilation in the ICU, informed consent given | RRT | Ceftazidime | HPLC | NON-LIN | Not reported | No |
| 143 | Van Dalen 1987 | Netherlands | Prospective PK study | 64 | Adults on mechanical ventilation in the intensive-care department | RRT | Cefoxitine, cefuroxime, ceftazidime, ceftriaxone | HPLC | NON-LIN | Not reported | No |
| 144 | van der Werf 1997 | Netherlands | Prospective PK study | 9 | Aged over 18 years; anuric renal failure; requiring CVVH; antimicrobial treatment including covering gram-negative pathogens, as judged by the attending physician; admitted to ICU; consent | RRT | Piperacillin/tazobactam | HPLC | KINFIT | Not reported | No |
| 145 | Vossen 2015 | Austria | Prospective PK study | 14 | Only sedated and intubated patients 18 years old or older and in need of ICU treatment and renal replacement therapy were deemed eligible | RRT | Doripenem | HPLC-UV | NONMEM 7.3 | Not reported | Yes |
| 146 | Wallenburg 2021 | Netherlands | Prospective PK study | 33 | ICU patients treated with flucloxacillin; ≥18 years; managed with CVC or arterial catheter | RRT | Flucloxacillin | LC-MS/MS | NONMEM 7.4.1 | Physiological plausibility | Yes |
| 147 | Wallenburg 2022 | Netherlands | Prospective PK study | 39 | ICU patients treated with piperacillin–tazobactam as part of routine clinical care; ≥ 18 years of age; started piperacillin–tazobactam therapy ≤ 72 h before inclusion; managed with CVC and/or arterial catheter | No | Piperacillin/tazobactam | UPLC-MS/MS | NONMEM 7.4.1 | Physiological plausibility | Yes |
| 148 | Westermann 2021 | Germany | Prospective PK study | 25 | Patients aged ≥18 years with sepsis treated with meropenem were eligible for study enrolment | RRT | Meropenem | LC-MS/MS | NONMEM 7.4 | Biological plausibility | Yes |
| 149 | Young 1997 | China | Prospective PK study | 10 | Adult patients in the Intensive Care Unit who required ceftazidime according to usual clinical practice were enrolled | No | Ceftazidime | HPLC | MK MODEL 5.02 | Not reported | Yes |
| 150 | Zahr 2022 | France | Retrospective PK study | 55 | Hospitalized in the ICU. Patients were treated by cefiderocol in combination with other antibiotics for ventilator-associated bacterial pneumonia | No | Cefiderocol | UPLC-MS/MS | Monolix 2020R1 | Not reported | Yes |
| 151 | Zhao 2022 | China | Prospective PK study | 64 | Patients admitted to the hospital with a diagnosis of pneumonia; use of meropenem empirically or therapeutically; time of continuous medication > 2 days; at least one steady-state plasma concentration could be obtained; age > 18 years; gram-negative bacteria isolated from specimen culture if possible | No | Meropenem | HPLC | Phoenix NLME 8.1 | Not reported | Yes |

*AKI, acute kidney injury; APACHE,* *Acute Physiology and Chronic Health Evaluation Score; ARC, augmented renal clearance; BMI, body mass index; CLCr, creatinine clearance; CVC, central venous catheter; CVVH, continuous veno-venous haemofiltration; CVVHD, continuous veno-venous haemodialysis; CVVHDF, continuous veno-venous haemodiafiltration; ECMO, extracorporeal membrane oxygenation; EDDf, extended daily dialysis with filtration; EVD, extraventricular drain; GFR, glomerular filtration rate; HAP, hospital-acquired pneumonia; HILIC-MS, hydrophilic interaction chromatography with mass spectrometry; HPLC-UV, high-performance liquid chromatography with UV spectroscopy; HPLC, high-performance liquid chromatography; IBW, ideal body weight; ICU, intensive care unit; IDMS, isotope dilution mass spectrometry; LC-MS/MS, liquid chromatography with tandem mass spectrometry; LRT, likelihood ratio test; MDR, multi-drug resistant; MDRD, Modification of Diet in Renal Disease; MODS, multi-organ dysfunction syndrome; MSSA, methicillin-sensitive Staphylococcus aureus; PK, pharmacokinetic; RRT, renal replacement therapy; SLED, sustained low-efficiency dialysis; TBSA, total body surface area; TBW, total body weight; TDM, therapeutic drug monitoring UHPLC-MS/MS, ultra-high performance liquid chromatography with tandem mass spectrometry; UHPLC, ultra-high performance liquid chromatography; UPLC-MS, ultra-high performance liquid chromatography with mass spectrometry; USA, United States of America; UTI, urinary tract infection; VA, veno-arterial; VAP, ventilator-associated pneumonia; VV, veno-venous; WBC, white blood cell*

**Figure S1: PRISMA 2020 Checklist**

**Figure S2: SWiM Checklist**

**Figure S3: ClinPK reporting quality assessments by study**


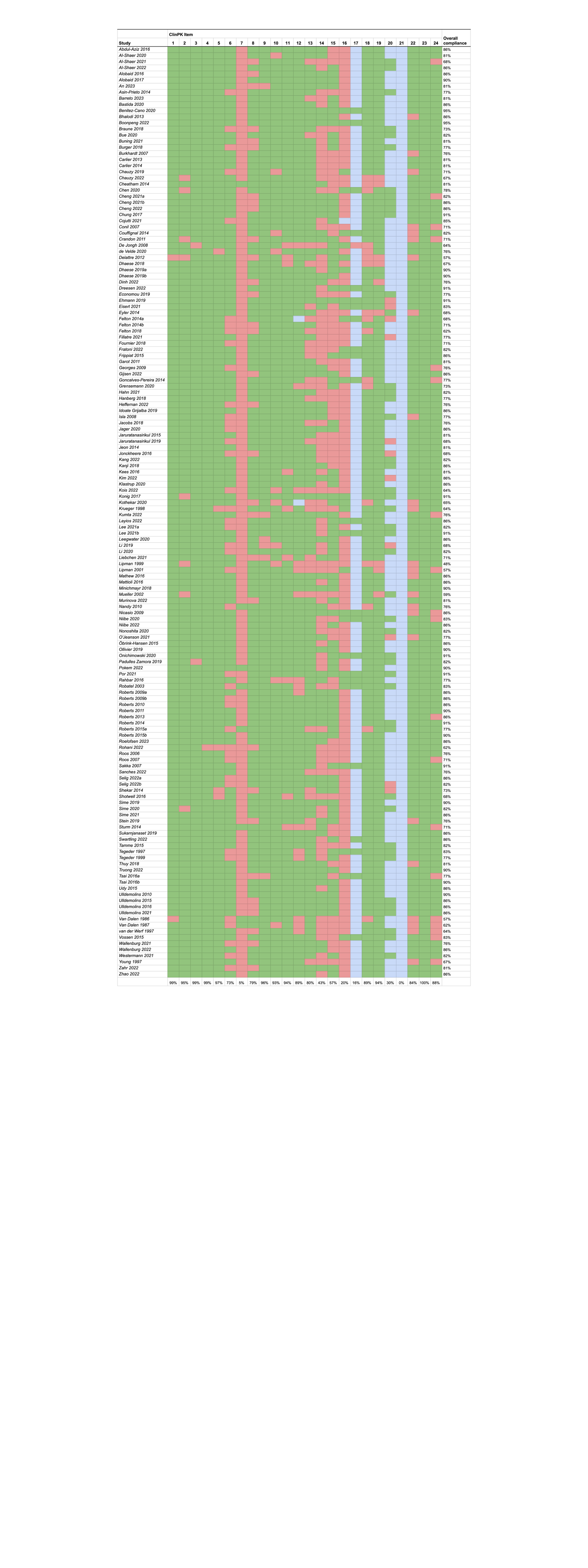


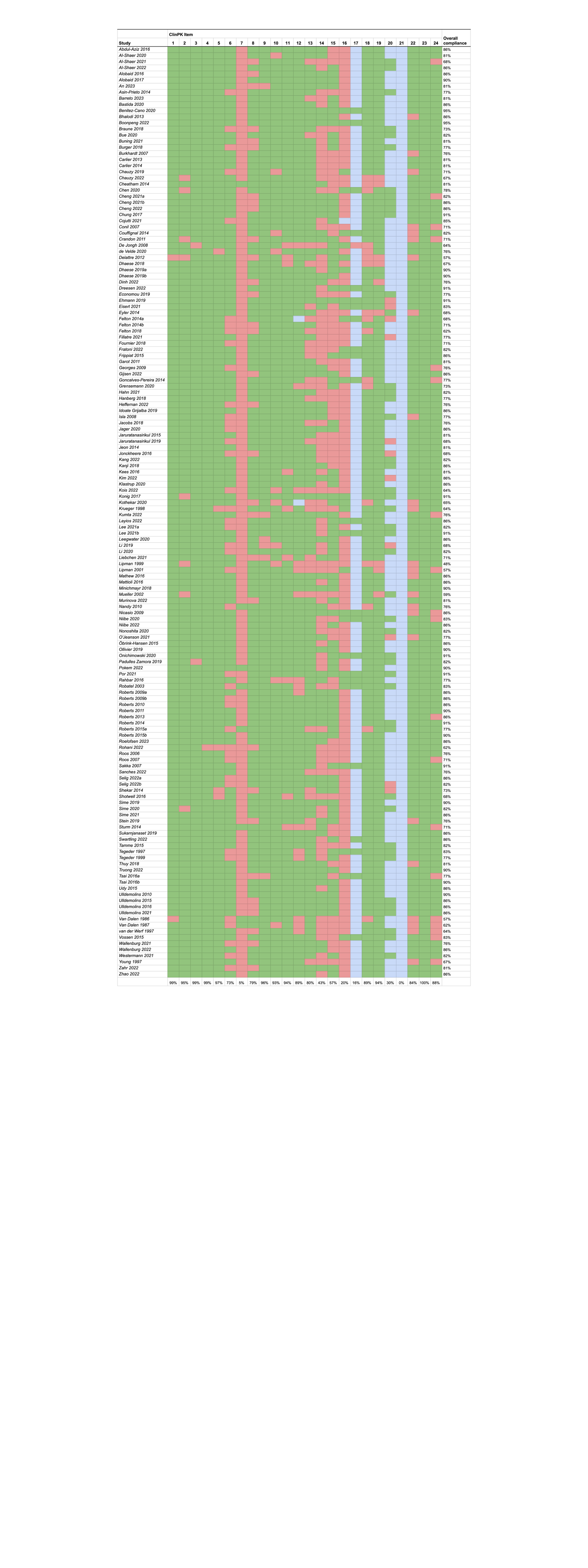


Figure S3 ClinPK reporting quality assessment for each included study

*Green, yes; red, no; blue, not applicable.*

**Appendix S2-3: Risk of Bias 2 Assessments**

Figure S4 Risk of bias summary

Review authors' judgements about each risk of bias item for each included randomised controlled trial.

Figure S5 Risk of bias graph

Review authors’ judgements about each risk of bias item presented as percentage across all included randomised controlled trials.

**Figures S6-8: Heatmaps of covariates reported in more than one study for each antimicrobial studied**


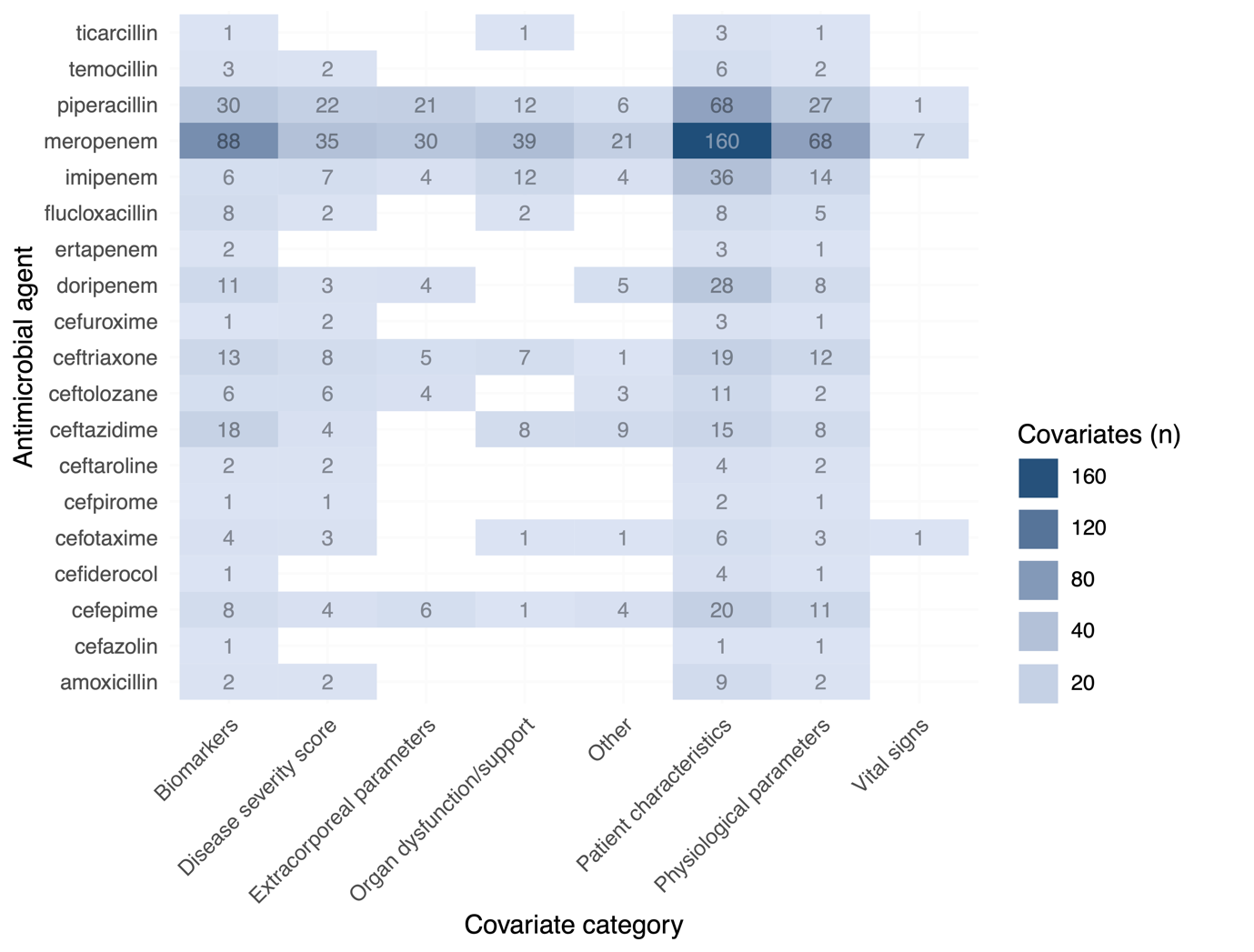


Figure S6 Heatmap of reported covariate categories according to antimicrobial studied

Of the 1083 covariates identified, studies using meropenem as the primary antimicrobial represented 441 (41%). Piperacillin (most often in combination with tazobactam) was assessed with 174 (16%) covariates in studies included in the final review. Ceftriaxone and ceftazidime were the most commonly evaluated cephalosporin antimicrobials.


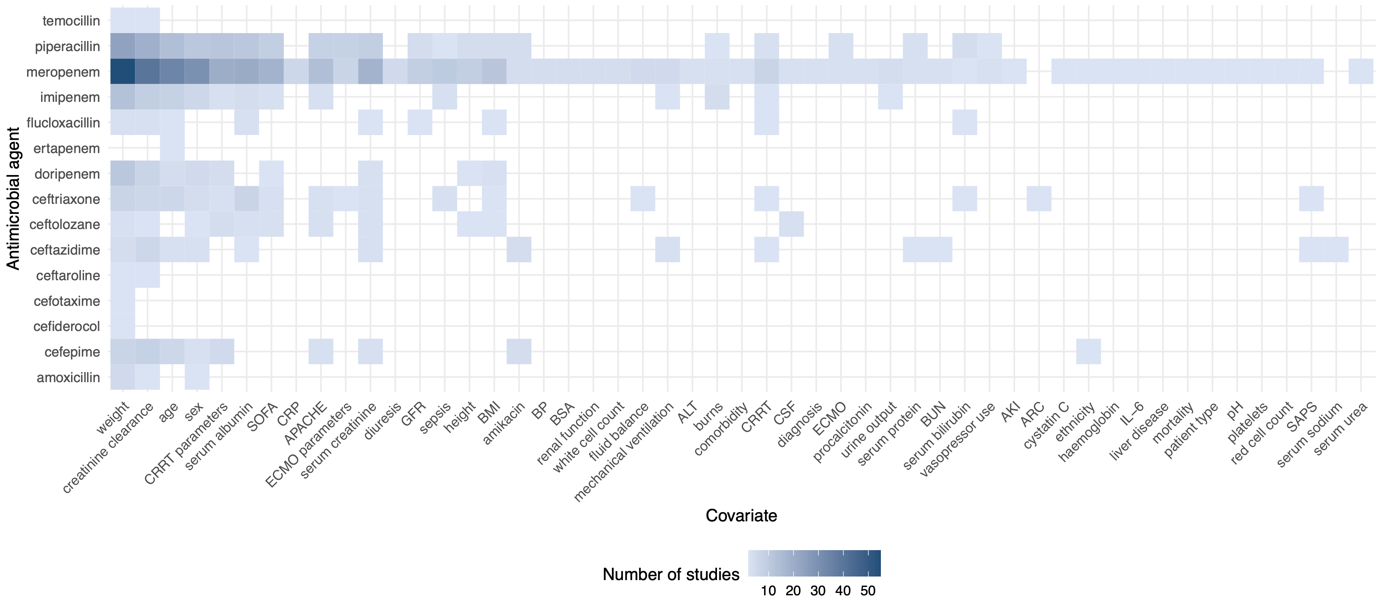


Figure S7 Heatmap of covariates reported in more than one study for each antimicrobial studied


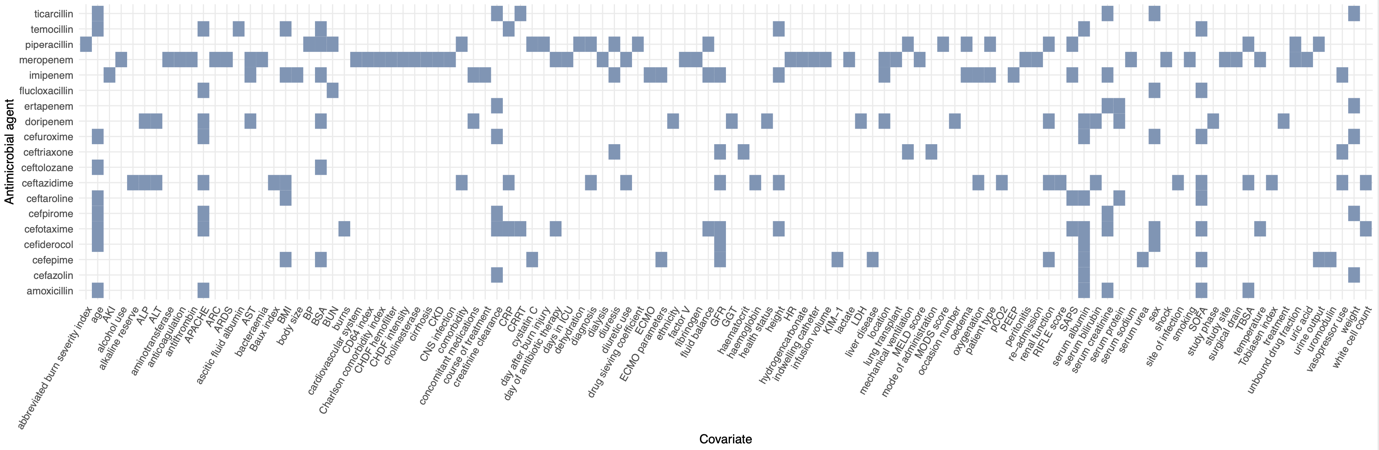


Figure S8 Heatmap of covariates reported in one study only for each antimicrobial studied
